# Causality between immunocytes and polymyositis: A Mendelian randomization analysis

**DOI:** 10.1097/MD.0000000000040254

**Published:** 2024-10-25

**Authors:** Ni Yang, Chang Li, Ruhui Liu, Xianghua Qi, Xing Qian

**Affiliations:** a Department of First Clinical Medical College, Shandong University of Traditional Chinese Medicine, Jinan, China; b Qingdao Haici Traditional Chinese Medicine Medical Group North Campus (Qingdao Hongdao People’s Hospital), Preventive Medicine Department, Jinan, China; c Affiliated Hospital of Shandong University of Traditional Chinese Medicine, Jinan, China.

**Keywords:** causality association, immunocytes, Mendelian randomization, polymyositis

## Abstract

Polymyositis is a prominent subgroup of idiopathic inflammatory myopathy, considered to have an autoimmune etiology. However, research exploring the condition between immunocytes and polymyositis remains limited, indicating the need for further investigation to unravel these intricate associations. We employed bidirectional Mendelian randomization (MR) analysis to ascertain causality between 731 immunocytes and polymyositis. We also compared the positive immunocytes with dermatomyositis. Our primary analytical method was inverse variance weighted, supplemented by 4 other MR techniques. Additionally, Cochran *Q* test was performed to assess heterogeneity, MR-Egger to appraise pleiotropy, and MR-PRESSO to identify and eliminate potential outliers. Furthermore, the leave-one-out test evaluated the impact of each instrumental variable (IV) on the causal effect. The inverse variance weighted results revealed that 10 immunocytes exert a protective effect against polymyositis (*P* < .05, OR < 1), while 16 immunocytes are connected with an elevated risk of the disease (*P* < .05, OR > 1). In reverse MR, polymyositis was found to decrease the levels of 2 immune cells (*P* < .05, OR < 1) and elevate the expression of 5 immune cell phenotypes (*P* < .05, OR > 1). A complex correlation was found between polymyositis and the immunocyte phenotypes CD8, CD33dim, HLA-DR, CD11b, and CD45. Additionally, it was discovered that 15 types of immune cells share a causal relationship between polymyositis and dermatomyositis. All analyses demonstrated no heterogeneity or horizontal pleiotropy (*P* > .05). Our study provides compelling evidence regarding the intricate causal relationships between immunocytes and polymyositis. Polymyositis and dermatomyositis share common immunocytes’ regulatory mechanisms. CD8, CD33dim, HLA-DR, CD11b, and CD45 may represent potential immune cell markers for polymyositis. These findings hold implications for planning prognosis and therapeutic strategies for polymyositis, offering novel insights for drug development.

## 1. Introduction

Polymyositis, a prominent subset of idiopathic inflammatory myopathy with presumed autoimmune etiology, primarily affects adults, and targets systemic skeletal muscles.^[1]^ Its clinical presentation typically includes symmetrical proximal limb and trunk muscle weakness of varying severity, elevated muscle-related kinases persisting for weeks to months, often accompanied by extra-muscular complications such as interstitial lung diseases, arthritis, and malignancies.^[2]^ With an incidence rate of 2 per 100,000 individuals, polymyositis predominantly affects women.^[3]^ However, due to its rarity and lack of distinct clinical features, misdiagnosis is common.^[4]^ Histologically, polymyositis is characterized by fiber size variability and scattered necrotic and regenerating fibers. Moreover, the identification of 2 different infiltrates of inflammatory cellular has suggested the participation of 2 immune-mediated pathways in the inflammation of muscle fibers: one contains infiltrates that are mainly located in the endomysium around the non-necrotic muscle fibers, known as endomysial infiltrates, and the other involves perimysium surrounding blood vessels, named perivascular infiltrates.^[5]^ Macrophages and activated CD8+ cytotoxic T lymphocytes make up the majority of these infiltrates; non-necrotic muscle fibers expressing major histocompatibility complex class I are occasionally invaded as well.^[6]^ Initial reports employing immunohistochemistry to characterize inflammatory cells in muscle biopsies of myositis individuals revealed the presence of CD4+ and CD8+ T cells, as well as B cells within the inflammatory infiltrates, suggesting a role for adaptive immunity in pathogenesis.^[7]^

The precise pathogenesis of polymyositis remains elusive, although external factors such as medications, infections, and malignancies, in conjunction with genetic predispositions, are believed to contribute.^[8]^ Research indicates a heightened susceptibility to polymyositis among individuals with specific human leukocyte antigen profiles.^[9]^ The presence of various immune cells including T cells, macrophages, dendritic cells, B cells, and plasma cells within muscle tissue, along with autoantibodies detected in up to 80% of patients, suggests an immune-mediated pathogenesis, which significantly implicates therapeutic approaches.^[10,11]^ Glucocorticoids are commonly used as first-line therapy, albeit their utility is constrained by frequent adverse effects.^[5]^ Combining glucocorticoids with steroid-sparing immunosuppressive agents can mitigate initial glucocorticoid dosages required for inducing remission, reduce the recurrence risk in the period of glucocorticoid tapering, and alleviate glucocorticoid-related adverse effects.^[6]^ However, the quest for treatment modalities for polymyositis with minimal side effects and proven efficacy remains ongoing. Therefore, further investigation is warranted to unravel the intricate interplay between immunocytes and polymyositis, elucidating disease mechanisms and laying the groundwork for adjunctive diagnosis and potential therapeutic interventions.

Genome-wide association studies (GWAS) are instrumental in analyzing genetic variations across large cohorts, pinpointing genetic positioning, and enriching our comprehension of the intricate genetic determinants of diseases.^[12]^ Mendelian randomization (MR) analysis leverages genetic variations sourced from GWAS databases to evaluate observed causal relationships.^[13]^ Unlike traditional observational studies, MR proves robust in identifying causal connections between instrumental variables (IVs) and diseases.^[14]^ Therefore, our objective is to elucidate the causality between immunocytes and polymyositis through bidirectional MR analysis, facilitating comprehension of the intricate involvement of immune cells in polymyositis. In addition, since polymyositis and dermatomyositis are both idiopathic inflammatory myopathies with similar clinical manifestations,^[15]^ we also compared the immune cells with the same causal relationship between polymyositis and dermatomyositis to promote understanding of the immune regulatory mechanisms between these 2 diseases.

## 2. Materials and methods

### 2.1. Study design

**Figure 1.**
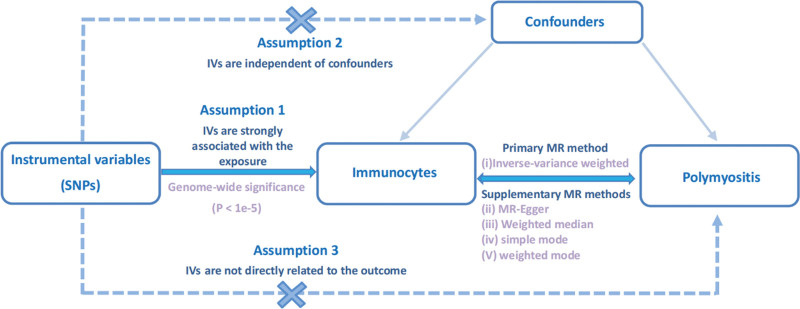
Overview of this bidirectional MR study design. MR = Mendelian randomization.

We explored the causal relationship between 7 categories of immunocytes and polymyositis using bidirectional MR analysis, which employs genetic variation as the proxy for risk factors. This necessitates valid IVs that adhere to 3 crucial assumptions: (1) IVs selected should exhibit a strong association with immunocytes (or polymyositis in reverse MR analysis); (2) there should be no association between IVs and the occurrence of the outcome or any other potential confounders linking immunocytes and polymyositis; and (3) IVs should not affect the outcome by means of paths other than exposure.^[16]^ Our research followed the STROBE principle (Supplementary File 1, Supplemental Digital Content, http://links.lww.com/MD/N813). The overall design is depicted in Figure 1.

### 2.2. Sources of GWAS data for polymyositis and immunocyte phenotypes

The investigation on immunocytes in this study drew upon GWAS data, encompassing more than 20 million variants across 731 immunocyte characteristics observed in 3757 individuals from Sardinia.^[17]^ Flow cytometry was employed to analyze the relative counts of 539 immune traits, encompassing 118 absolute cell counts (AC), 389 median fluorescence intensity values of surface antigens, and 32 morphological parameters. It also examined relative counts, comprising 192 levels of cells in peripheral blood using flow cytometry. The cell traits were classified into different panels within the database, including mature B cell panel, dendritic cell (DC) panel, mature T cell panel, monocyte panel, myeloid cell panel, TBNK cell panel (T cells, B cells, and NK cells), and Treg panel. Genome-wide interpolation and adjustment for multiple tests were performed concerning the Sardinian sequence and Minimac software.

The GWAS statistics concerning polymyositis were derived from the FinnGen R10 database, encompassing 244 patients (150 females) and 399,355 healthy individuals. All patients met the diagnostic criteria for polymyosis in ICD-10M33.2, and the median age at the first event was 55.94 years old.

The GWAS statistical data on dermatomyositis comes from the FinnGen R11 database, which includes 459 patients (282 females) and 341,375 healthy participants. All patients met the diagnostic criteria for dermatomyositis in ICD-10M33, with a median age of 56.07 years at first onset.

### 2.3. Screening of IVs

IVs were considered significant if their association with each phenotype reached a threshold of 1 × 10^‐5^, while adhering to corresponding linkage disequilibrium arguments of r^2^ < 0.001 and kb = 10,000.^[18]^ The LD Trait Tool on the LDlink website was utilized to validate the inclusion of single nucleotide polymorphism (SNP) loci and to exclude potential confounders associated with the SNPs, such as estrogen receptor + breast cancer.^[19]^ Additionally, palindromic SNPs were excluded, and the significance of IVs was further confirmed by selecting SNPs with F-statistics exceeding 10.

### 2.4. Statistical analysis

The TwoSampleMR package in R 4.3.2 conducted our analysis, employing 5 common methods: inverse variance weighted (IVW),^[20]^ weighted median, simple mode,^[21]^ weighted mode,^[22]^ and MR-Egger regression, with IVW as the major analytical method supplemented by others. Sensitivity analyses were performed to address potential pleiotropy.

Heterogeneity was assessed using IVW(Q) and MR-Egger(Q), while horizontal pleiotropy was explored through the intercept measurement of MR-Egger.^[23]^ The MR pleiotropic residuals and outliers method^[24]^ (NbDistribution = 10,000) was employed to identify potential outliers in MR analysis.^[23]^ After outlier removal, heterogeneity was reassessed, and the influence of each SNP on the general outcomes was evaluated using leave-one-out analysis. *P* < .05 was considered statistical significance.

## 3. Results

### 3.1. Causal effects of immunocytes on polymyositis

In this investigation, the GWAS data of 731 immunocyte phenotypes were screened for IVs, all exhibiting F-statistics exceeding 10, thus eliminating concerns regarding weak instrumental variable bias. Detailed information about the SNPs identified as positive IVs is provided in Table S1, Supplemental Digital Content, http://links.lww.com/MD/N816

**Figure 2.**
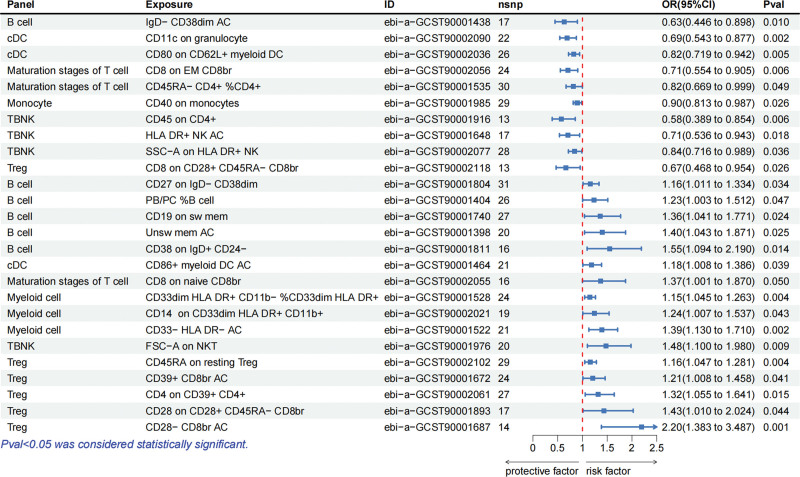
Forest plot showed the causal effects of immunocytes on polymyositis. Panel = the included immunocytes were divided into different panels, including B cell panel (mature B cell panel), cDC panel (dendritic cell panel), maturation stages of T cell panel, monocyte panel (single-cell panel), myeloid cell panel, TBNK panel (T cells, B cells, and NK cells), and Treg panel; Exposure = the specific types of immunocytes; ID = specific types of immunocytes numbered in the database; nsnp = the number of SNPs included.

Figure 2 illustrates the results obtained through the genetically predicted IVW method for 7 categories of immune cells about polymyositis. Notably, 10 immune cell traits demonstrated protective roles in the occurrence of polymyositis (OR < 1, *P* < .05): B cell panel: IgD‐ CD38dim AC; cDC panel: CD11c on granulocyte and CD80 on CD62L+ myeloid DC; maturation stages of T cell panel: CD8 on effector memory CD8+ T cell and CD45RA‐ CD4+ %CD4+; monocyte panel: CD40 on monocytes; TBNK panel: CD45 on CD4+, HLA-DR+ NK AC, and SSC-A on HLA-DR+ NK; Treg panel: CD8 on CD28+ CD45RA‐ CD8+ T cell. While 16 traits exhibited a correlation with increased polymyositis development (OR > 1, *P* < .05): B cell panel: CD27 on IgD‐ CD38dim, PB/PC %B cell, CD19 on switched memory B cell, unswitched memory B cell AC, and CD38 on IgD+ CD24‐; cDC panel: CD86+ myeloid DC AC; maturation stages of T cell panel: CD8 on naive CD8+ T cell; myeloid cell panel: CD33dim HLA-DR+, CD11b‐ %CD33dim HLA-DR+, CD14 on CD33dim HLA-DR+ CD11b+, and CD33‐ HLA-DR‐ AC; TBNK panel: FSC-A on NKT; Treg panel: CD45RA on resting Treg, CD39+ CD8+ T cell AC, CD4 on CD39+ CD4+, CD28 on CD28+ CD45RA‐ CD8+ T cell, and CD28‐ CD8+ T cell AC. Table S2, Supplemental Digital Content, http://links.lww.com/MD/N816 presents the outcomes derived from 5 MR analysis methods, and Supplementary File 2, Supplemental Digital Content, http://links.lww.com/MD/N814 includes scatter plots corresponding to the 26 data points above.

**Figure 3.**
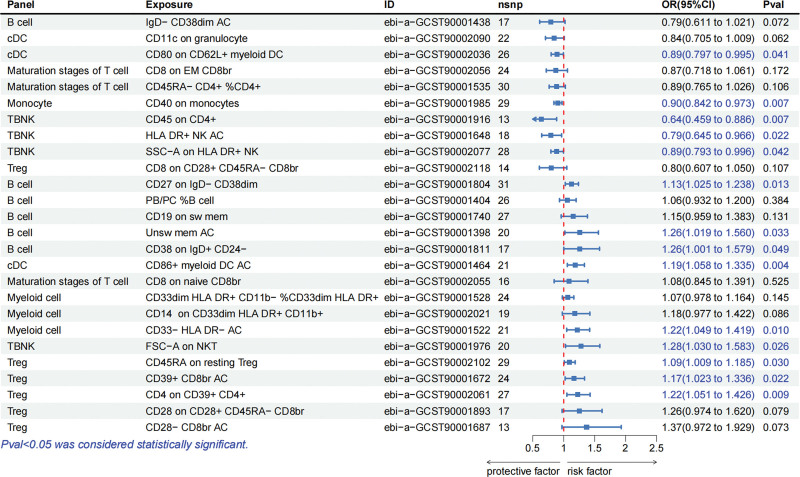
Forest plot showed the causal effects of immunocytes on dermatomyositis. Panel = the included immunocytes were divided into different panels, including B cell panel (mature B cell panel), cDC panel (dendritic cell panel), maturation stages of T cell panel, monocyte panel (single-cell panel), myeloid cell panel, TBNK panel (T cells, B cells, and NK cells), and Treg panel; Exposure = the specific types of immunocytes; ID = specific types of immunocytes numbered in the database; nsnp = the number of SNPs included.

Among the positive results mentioned above, 14 immune cell phenotypes showed the same causal relationship in dermatomyositis (Fig. 3). In detail, CD80 on CD62L+ myeloid DC in the cDC panel, CD40 on monocytes in the monocyte panel, CD45 on CD4+, HLA-DR+ NK AC, and SSC-A on HLA DR+ NK in the TBNK panel have protective effects on both diseases. However, CD27 on IgD− CD38dim, unswitched memory B cell AC, and CD38 on IgD+ CD24− in B cell panel, CD86+ myeloid DC AC in cDC panel, CD33− HLA-DR− AC in myeloid cell panel, FSC− A on NKT in TBNK panel, CD45RA on resting Treg, CD39+ CD8+ T cell AC, and CD4 on CD39+ CD4+ in Treg panel showed increased risks of developing both diseases.

### 3.2. Causal effects of polymyositis on immunocytes

Applying identical criteria, we incorporated 6 qualified SNPs connected with polymyositis as exposure IVs. Explicit information concerning eligible SNPs is available in Table S3, Supplemental Digital Content, http://links.lww.com/MD/N816.

**Figure 4.**
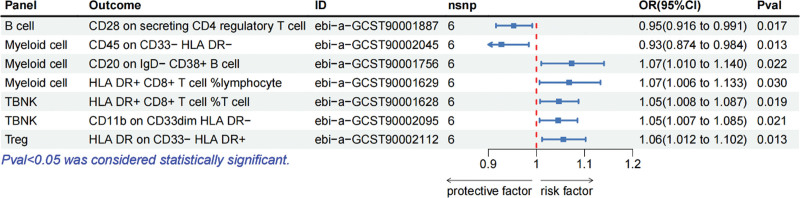
Forest plot showed the causal effects of polymyositis on immunocytes. Panel = the included immunocytes were divided into different panels, including B cell panel (mature B cell panel), cDC panel (dendritic cell panel), maturation stages of T cell panel, monocyte panel (single-cell panel), myeloid cell panel, TBNK panel (T cells, B cells, and NK cells), and Treg panel; Outcome = the specific types of immunocytes; ID = specific types of immunocytes numbered in the database; nsnp = the number of SNPs included.

Figure 4 illustrates the outcomes obtained through the genetically predicted IVW method regarding the impact of polymyositis on 7 categories of immune cells. Specifically, polymyositis demonstrated the capacity to decrease the levels of 2 immune cell types (OR < 1, *P* < .05): B cell panel: CD28 on secreting CD4 regulatory T cell and myeloid cell panel: CD45 on CD33‐ HLA-DR‐. However, polymyositis can simultaneously elevate the levels of another 5 immunocytes (OR > 1, *P* < .05): myeloid cell panel: CD45 on CD33‐ HLA-DR‐, CD20 on IgD‐ CD38+ B cell, and HLA DR+ CD8+ T cell %lymphocyte; TBNK panel: HLA-DR+ CD8+ T cell %T cell and CD11b on CD33dim HLA DR‐; Treg panel: HLA-DR on CD33‐ HLA DR+. After applying Bonferroni correction, none of the results achieved statistical significance. Table S4, Supplemental Digital Content, http://links.lww.com/MD/N816 presents the results derived from 5 MR analysis methods, with Supplementary File 3, Supplemental Digital Content, http://links.lww.com/MD/N815 containing scatter plots corresponding to the aforementioned 7 data points.

**Figure 5.**
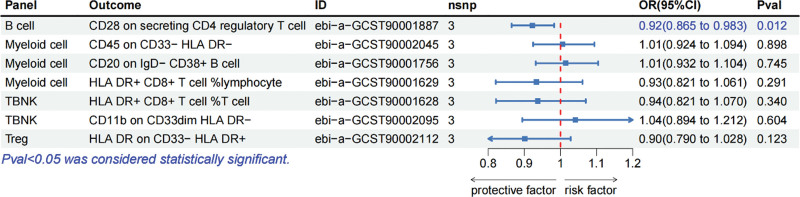
Forest plot showed the causal effects of dermatomyositis on immunocytes. Panel = the included immunocytes were divided into different panels, including B cell panel (mature B cell panel), cDC panel (dendritic cell panel), maturation stages of T cell panel, monocyte panel (single-cell panel), myeloid cell panel, TBNK panel (T cells, B cells, and NK cells), and Treg panel; Outcome = the specific types of immunocytes; ID = specific types of immunocytes numbered in the database; nsnp = the number of SNPs included.

Following the criteria, 3 qualified SNPs associated with dermatomyositis were included as exposure IVs (Table S5, Supplemental Digital Content, http://links.lww.com/MD/N816). We found that both polymyositis and dermatomyositis can reduce the expression of CD28 on secreting CD4 regulatory T cell in B cell panel (Fig. 5).

### 3.3. Sensitivity analysis

Sensitivity analyses showed no heterogeneity or horizontal pleiotropy for all positive results, confirming the credibility of causally robust findings (Tables S6–S9, Supplemental Digital Content, http://links.lww.com/MD/N816). Additionally, both the leave-one-out and funnel plots consistently suggested the reliability of the data (Supplementary Files 2 and 3, Supplemental Digital Content, http://links.lww.com/MD/N814 http://links.lww.com/MD/N815).

## 4. Discussion

Our study examined the causality between 731 immunocyte phenotypes and polymyositis using bidirectional MR analysis. Our findings revealed evidence suggesting the influence of 26 immunocytes on the pathogenesis of polymyositis, accompanying the abnormal production of 7 immune cells in the blood associated with polymyositis. 15 immunocytes have the same causal relationship between polymyositis and dermatomyositis. Besides, we discovered complex participation of immune cell markers CD8+ Tcell, CD33dim, HLA-DR, CD11b, and CD45 in polymyositis.

Our results indicated that 10 immune cell phenotypes exhibit a protective effect against polymyositis, while 16 are associated with increased risk. Among these, CD11c on granulocytes, CD40 on monocytes, and HLA-DR+ NK AC demonstrated protective effects against polymyositis. The constitutive activation of CD11c advances neutrophil maturation and its release under stable-state conditions.^[25]^ CD40 signaling speeds up T lymphocyte-dependent B cell multiplication and immunoglobulin isotype switching.^[26]^ Additionally, CD40 ligation on monocytes and dendritic cells enhances their survival, cytokine and enzyme secretion, monocyte tumoricidal activity, and nitric oxide synthesis.^[27]^ Furthermore, the interaction connecting CD40 and its ligand has significant implications for the function of antigen-presenting cells as well as T cells.^[28]^ HLA-DR+ NK cells are capable of producing proinflammatory cytokines, degranulating, and proliferating easily in reply to stimuli.^[29]^ The protective immune cell phenotype may exert its effects through these aforementioned mechanisms. Several immune cell phenotypes exhibited the ability to increase the risk of polymyositis, such as PB/PC %B cells, CD86+ myeloid DCs, and CD28 on Treg cells. The proportion of B cells in peripheral blood and spleen is expressed as PB/PC %B cells, it participates in immune regulation and controls the immune response intensity.^[30]^ Dendritic cells (DCs), particularly myeloid DCs, play a crucial role in T cell function and the connection between congenital and adaptive immunity. Plasmacytoid DCs circulating display an unformed phenotype featured with low CD86 expression, while myeloid DCs notably rise throughout chronic infection.^[31]^ Muscle infiltrates in polymyositis patients are mostly CD28null T cells,^[32]^ which exhibit cytotoxicity to autologous muscle cells.^[33]^ Additionally, the increase of CD8+ CD28 circulating T cells is characteristic of systemic inflammation in myositic dermatomyositis.^[34]^

We observed through reverse MR analysis that polymyositis can cause abnormalities in the production of 7 types of immune cells, with 2 showing decreases and 5 showing increases. For example, we noted a downregulation of CD28 expression on secreting CD4 regulatory T cells and an upregulation of CD20 on IgD‐ CD38+ B cells. CD28 is essential in the generation of regulatory T cells in the periphery, controlling their differentiation from CD4 non-Tregs via IL-2 production.^[35]^ On the other hand, CD20, by associating with the B cell receptor,^[36]^ functions as an ion channel,^[37]^ and may trigger intracellular signaling pathways resulting in cell cycle arrest, homotypic adhesion, apoptosis, or even lysosome-mediated cell death.^[38–41]^ Rituximab, a chimeric monoclonal antibody targeting CD20, has been utilized in treating refractory inflammatory myopathies,^[42]^ yet the precise mechanism of its action warrants further investigation.

Furthermore, our study reveals the possible complex involvement of CD8+ T cell, CD33dim, HLA-DR, CD11b, and CD45 in polymyositis. Notably, an elevated presence of CD8 on naive CD8+ T cell is linked to an increased risk of polymyositis, whereas CD8 on Effector Memory CD8+ T cell demonstrates a protective effect against the disease. CD8+ T cells act as an essential part of adaptive immunity, engaging in the combat and regulation of various malignancies along with intracellular infections.^[43]^ Naive CD8+ T cells’ activation triggers a comprehensive program of proliferation and differentiation, yielding effector and memory CD8+ T cells.^[44]^ Effector memory T cells, characterized by immediate effector function,^[45]^ typically exhibit tissue-specific homing markers and integrins.^[46]^ In a steady state, it can enter nonlymphoid tissues through circulation,^[47]^ facilitating their recruitment to infection sites for rapid control.^[48]^ Studies have demonstrated that juvenile dermatomyositis syndrome is marked by a significant reduction in circulating CD8+ T cells, while these cells are notably infiltrated within interfascicular muscle tissue and around blood vessels.^[49]^ These infiltrating CD8+ T cells target major histocompatibility complex class I-expressing muscle cells by secreting cytotoxic effector molecules in conditions such as polymyositis and inclusion-body myositis.^[50]^ These findings align to some extent with our results. Our research has identified potential roles for various CD8+ T cell subsets, including effector memory CD8+ T cells, CD8+ CD28+ CD45RA‐ T cells, naive CD8+ T cells, CD28+ CD45RA‐ CD8+ T cells, and CD28‐ CD8+ T cells in polymyositis, though the specific mechanisms underlying these roles remain to be further investigated. Moreover, CD33dim, HLA-DR, and CD11b represent progranulocytes, late myelocytes, and proto-single cells, respectively, derived from normal myeloid cell lines.^[51]^ HLA-DR expression in myofibers serves as a typical biomarker for anti-synthetase-related myopathy,^[52]^ while in polymyositis, muscle fibers produce and express HLA-DR molecules, potentially contributing to inflammatory responses alongside lymphocytes.^[53]^ Immunohistochemical staining in experimental autoimmune myositis indicates muscle fiber infiltration involving CD11b+ cells.^[54]^ Additionally, elevated mRNA levels of CD11b in blood neutrophils are observed in amyopathic dermatomyositis with active interstitial lung disease.^[55]^ Furthermore, CD45 molecules on CD4+ T cells for mankind are utilized as markers of naive and memory T cells, with CD45 isoform expression transitioning CD45RA into CD45RO upon sensitization with particular internal antigens, and the sensitivity of CD4+ T cells to multiply spur appears to be partly regulated by CD45 isoform expression.^[56]^ Previous clinical observations identified that patients with recurrent lung adenocarcinoma receiving treatment with combined monoclonal antibodies often experience progressive neuromuscular ventilation deficits. This condition is characterized by atrophy of type II muscle fibers and inflammatory myopathy, which involves a mixed infiltration of CD8+ and CD4+ lymphocytes, suggesting potential targets for lymphocyte infiltration in polymyositis.^[57]^ The immune-mediated assault on skeletal muscle is primarily driven by CD8+ T cells, and CD4+ T cells also contribute to this process.^[58]^ Moreover, T cells lacking CD28, including both CD4+ and CD8+ subsets, have demonstrated cytotoxic effects on autologous muscle cells in patients with polymyositis.^[33]^ Nevertheless, the precise mechanisms governing these interactions warrant further investigation. Considering these findings, CD8+ T cell, CD33dim, HLA-DR, CD11b, and CD45 may represent potential immune cell markers for polymyositis, likely exerting their effects through the aforementioned mechanisms. However, the specific underlying mechanism remains unclear, necessitating further exploration.

Additionally, we have identified several novel findings in polymyositis, including the potential roles of IgD‐ CD38dim B cells, CD80 on CD62L+ myeloid dendritic cells, SSC-A on HLA DR+ natural killer cells, CD27 on IgD‐ CD38dim B cells, plasma blast–plasma cell %B cells, CD19 on switched memory B cells, unswitched memory B cells, CD38 on IgD+ CD24‐ B cells, CD14 on CD33dim HLA-DR+ CD11b+ cells, FSC-A on natural killer cells, CD39+ CD8+ T cell absolute count, and CD4 on CD39+ CD4+ T cells. These findings have not been previously reported and require further validation in clinical practice.

This study utilized bidirectional two-sample MR analyses, leveraging a substantial cohort from genomic studies characterized by robust statistical power and ample sample sizes. Our conclusions stem from a thorough exploration of genetic-level causal relationships, employing multiple MR analyses for both causal inference and result validation. As a result, the study’s findings maintain their robustness, remaining impervious to the influences of horizontal pleiotropy and confounding factors.

Nevertheless, our study encounters certain limitations. Primarily, our selection of IVs was predicated on a significance level of *P* < 1 × 10^‐5^, potentially incorporating IVs with weaker correlations albeit facilitating a more extensive evaluation of the connection between immunocyte phenotypes and polymyositis. Additionally, our dataset predominantly comprised European populations, prompting questions about the generalizability of our findings to other regional demographics and thereby constraining the applicability of our results. Furthermore, while we employed the *Q* test and Egger intercept to statistically assess and mitigate heterogeneity and horizontal pleiotropy, these methods do not offer absolute assurance of their absence in a clinical context. In addition, although our research results did not reach a significant level after Bonferroni correction, due to numerous classifications (731 immunocytes), this method also has the drawback of being too conservative, which may result in many “real” effects not being discovered.^[59]^ Therefore, our results provide a more comprehensive picture of the potential immune cells associated with polymyositis. Ultimately, comprehensive clinical trials are indispensable for elucidating the intricate causality between individual immunocytes and polymyositis, alongside their underlying mechanisms of influence.

## 5. Conclusion

Our research signified a causal link between various immunocytes and polymyositis utilizing a synthetical bidirectional two-sample MR analysis. This underscores the intricate interplay between the immunocyte phenotypes and polymyositis, in which CD8, CD33dim, HLA-DR, CD11b, and CD45 may represent potential immune cell markers for polymyositis, paving the way for novel avenues of research into the biological mechanisms of polymyositis and facilitating disease prevention and early treatment.

## Acknowledgments

Thanks are due to all participants in the IEU Open GWAS Project investigations, and the academic researchers for offering the publicly available data.

## Author contributions

**Conceptualization:** Ni Yang, Xianghua Qi.

**Data curation:** Xing Qian.

**Formal analysis:** Ni Yang, Chang Li.

**Investigation:** Chang Li, Ruhui Liu.

**Software:** Ni Yang, Chang Li.

**Validation:** Ni Yang.

**Writing – original draft:** Ni Yang.

**Writing – review & editing:** Chang Li, Xing Qian.

## Supplementary Material


