# Supplementary material for: Causality between immunocytes and polymyositis: A Mendelian randomization analysis
Source: Medicine (Baltimore). 2024 Oct 25;103(43):e40254. doi: 10.1097/MD.0000000000040254 (PMC11521033; doi:10.1097/MD.0000000000040254)
Supplement: Supplementary file 3 [file medi-103-e40254-s003.docx]

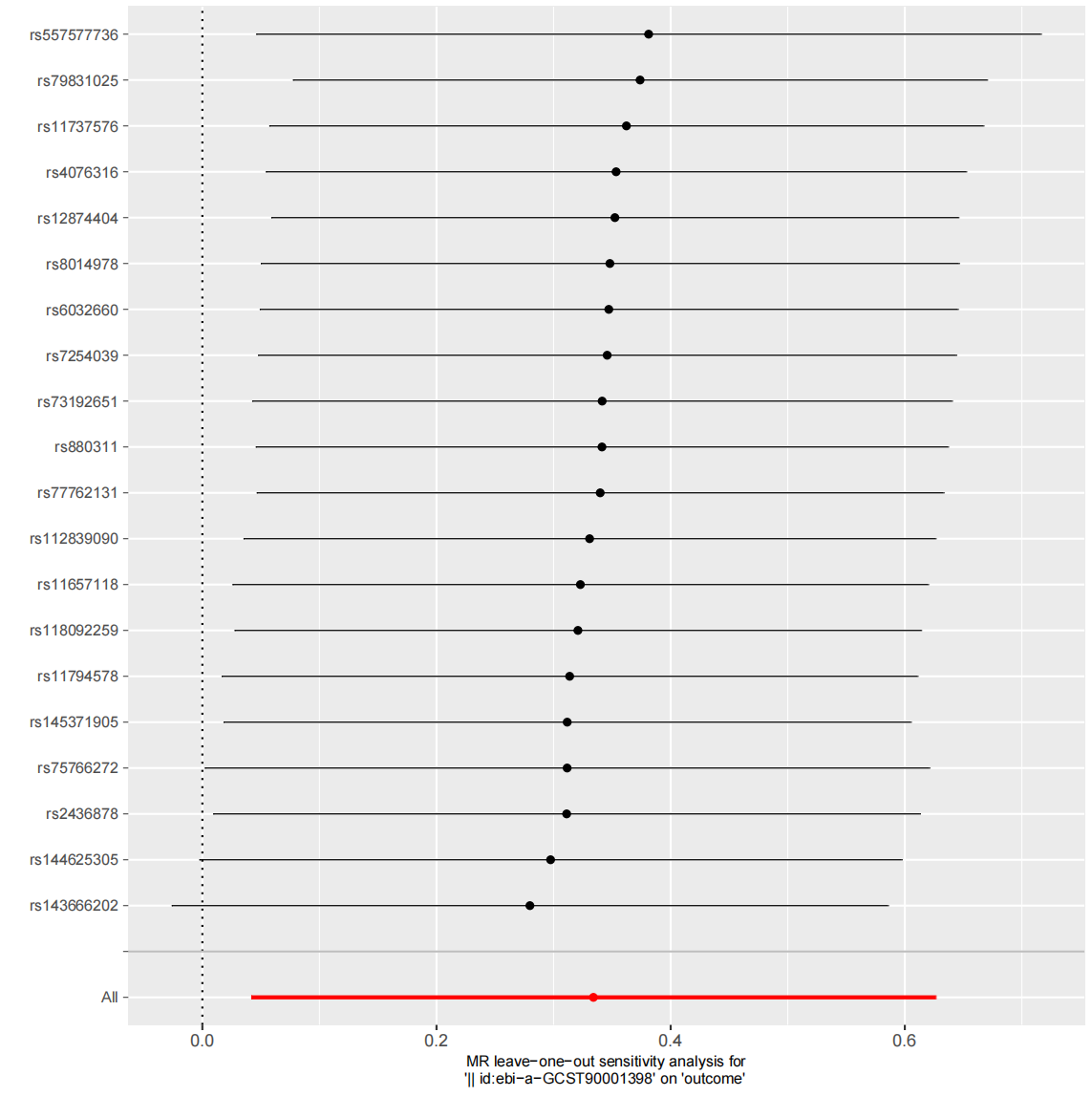


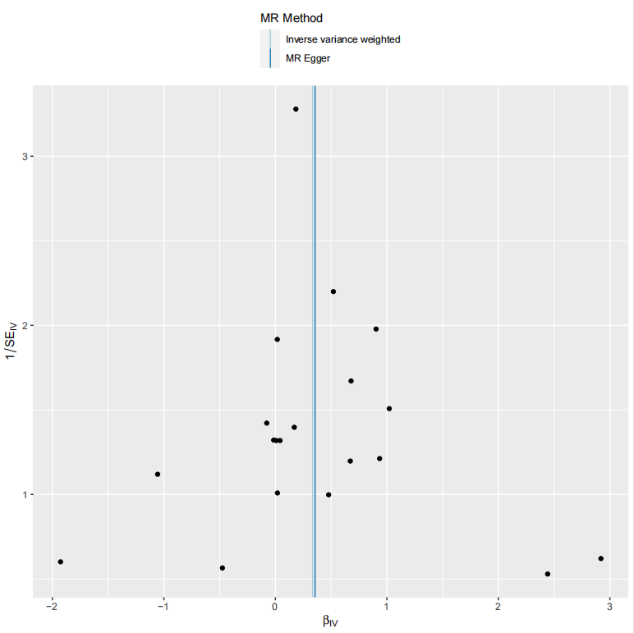

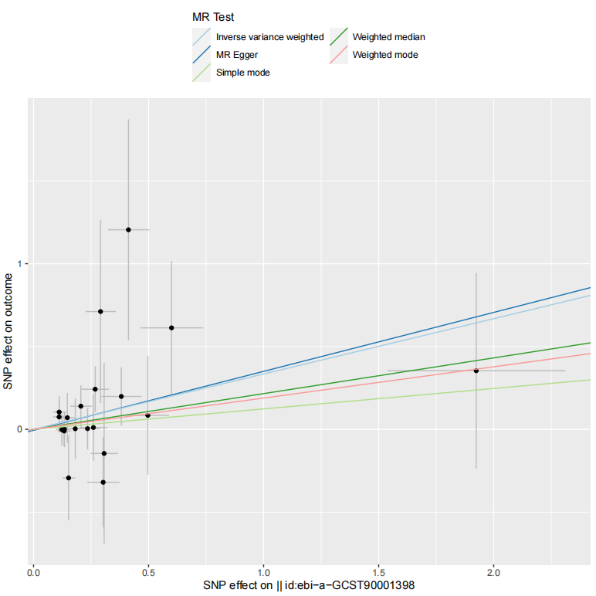


The leave-one-out, funnel, and scatter plots of the causal effect of Unsw mem AC on polymyositis.


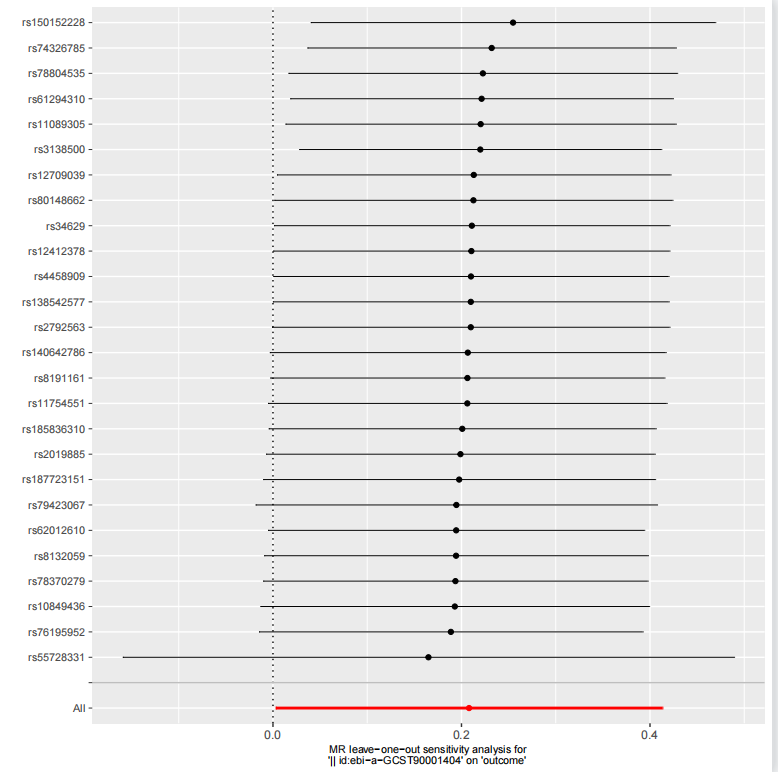


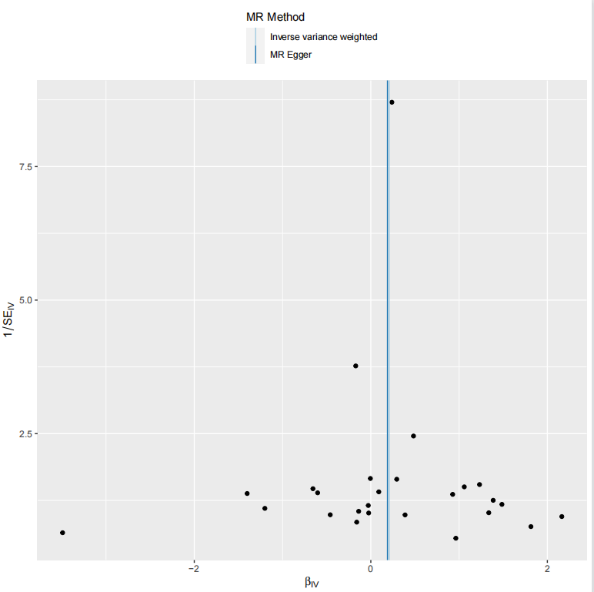

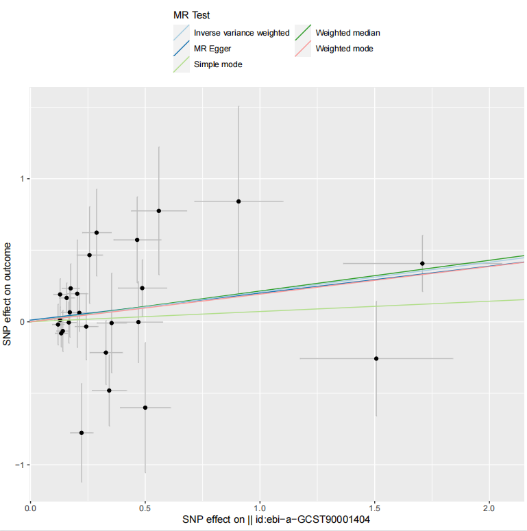


The leave-one-out, funnel, and scatter plots of the causal effect of PB/PC %B cell on polymyositis.


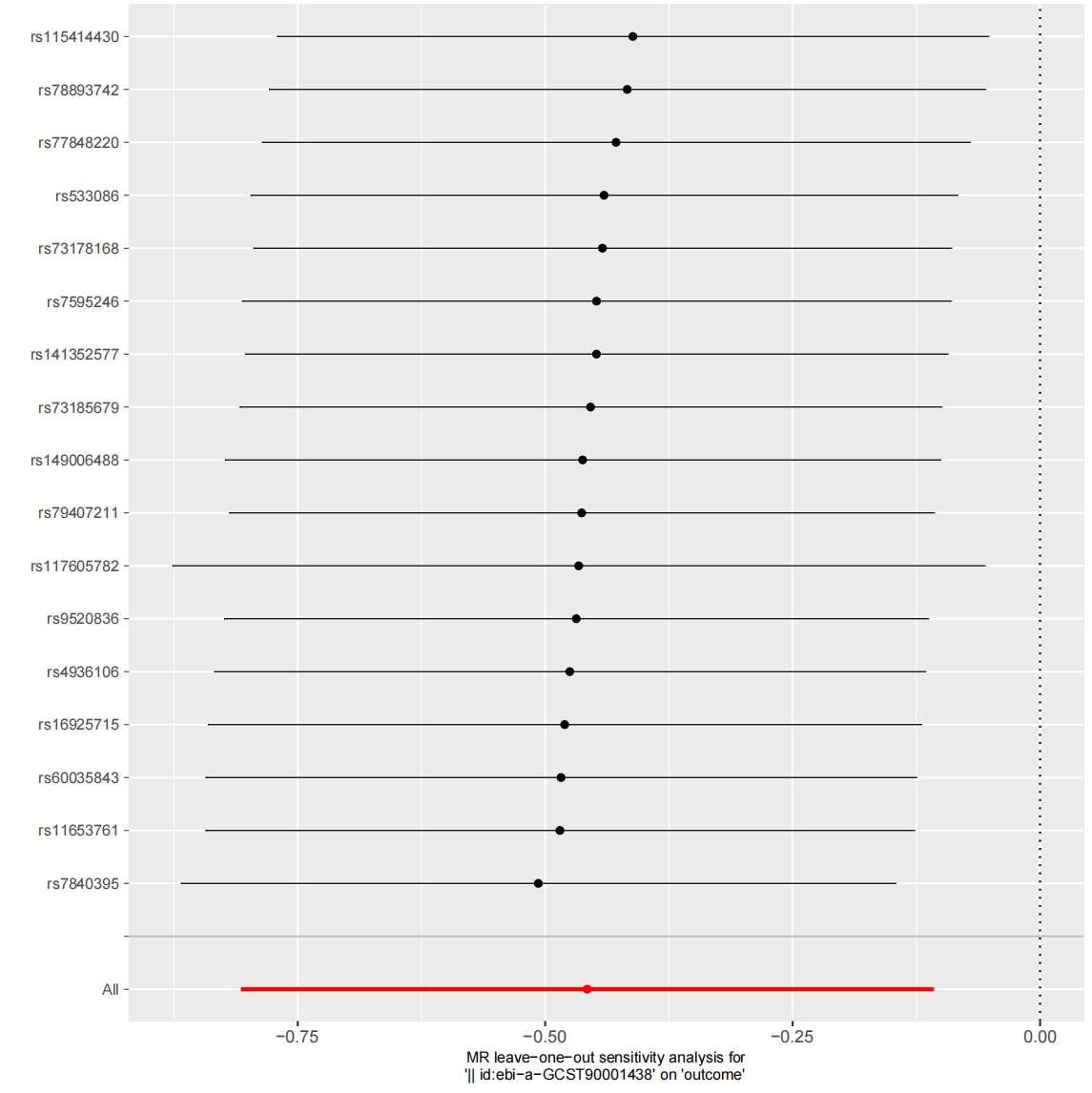


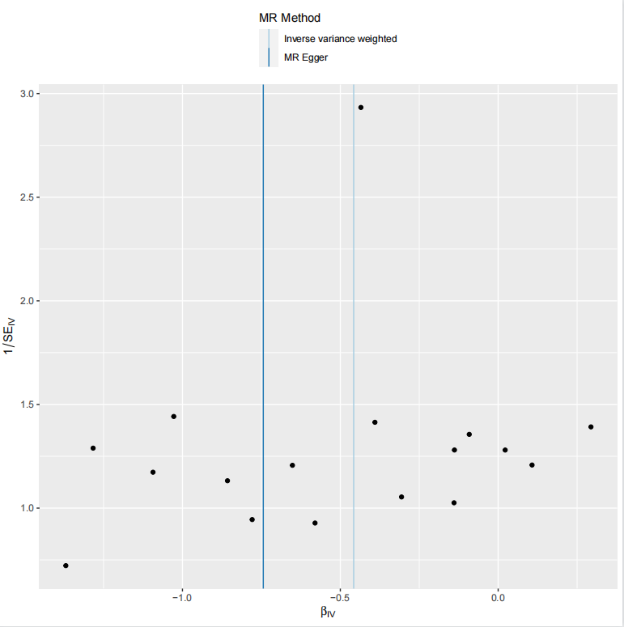

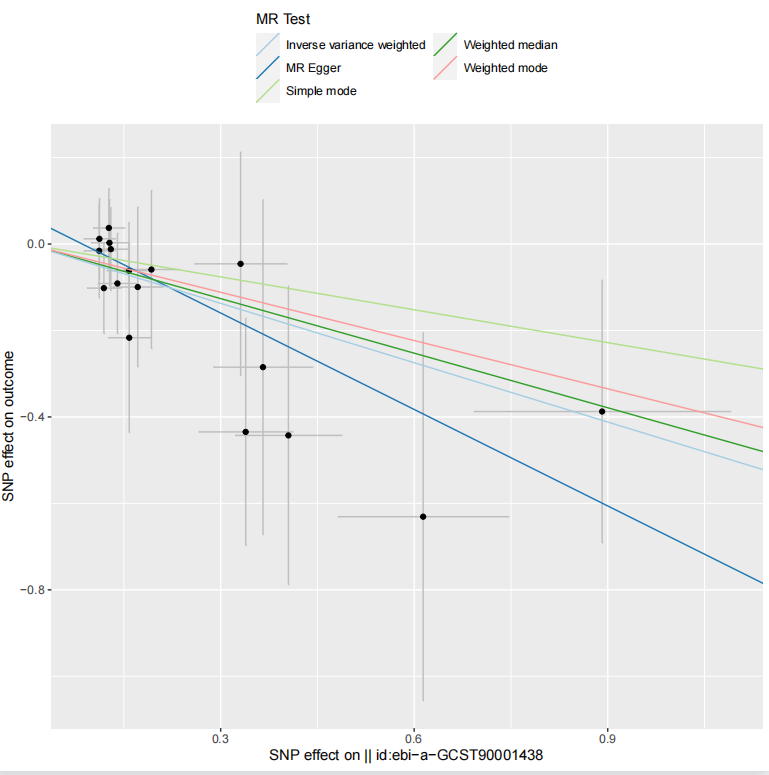


The leave-one-out, funnel, and scatter plots of the causal effect of IgD- CD38dim AC on polymyositis.


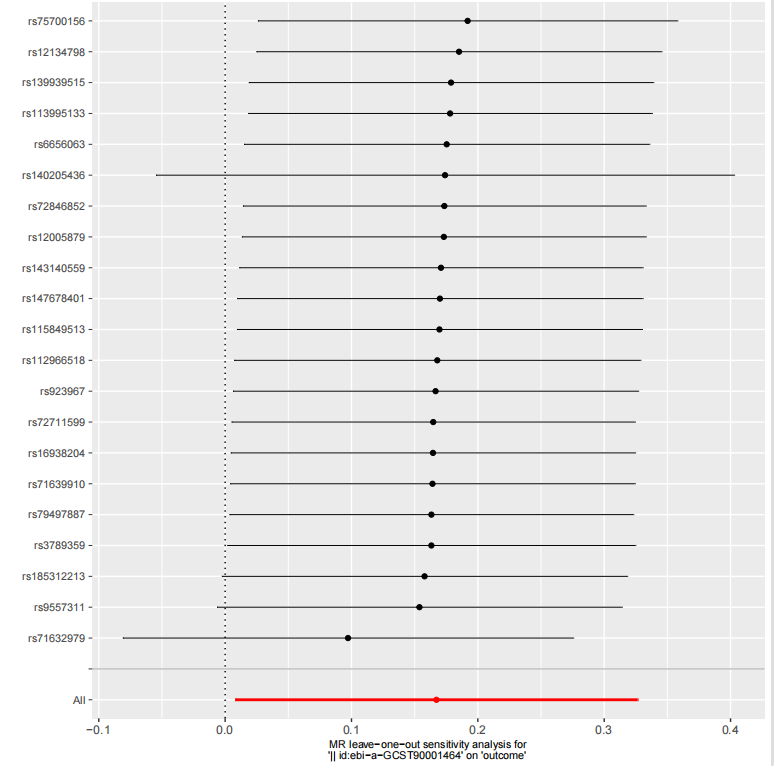


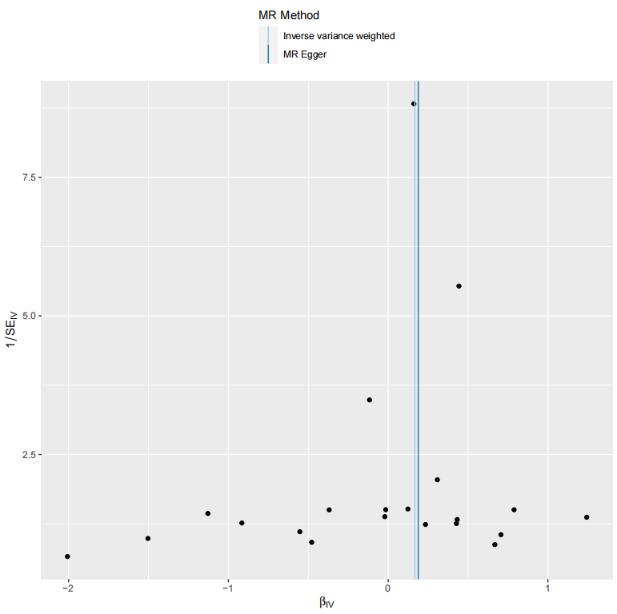

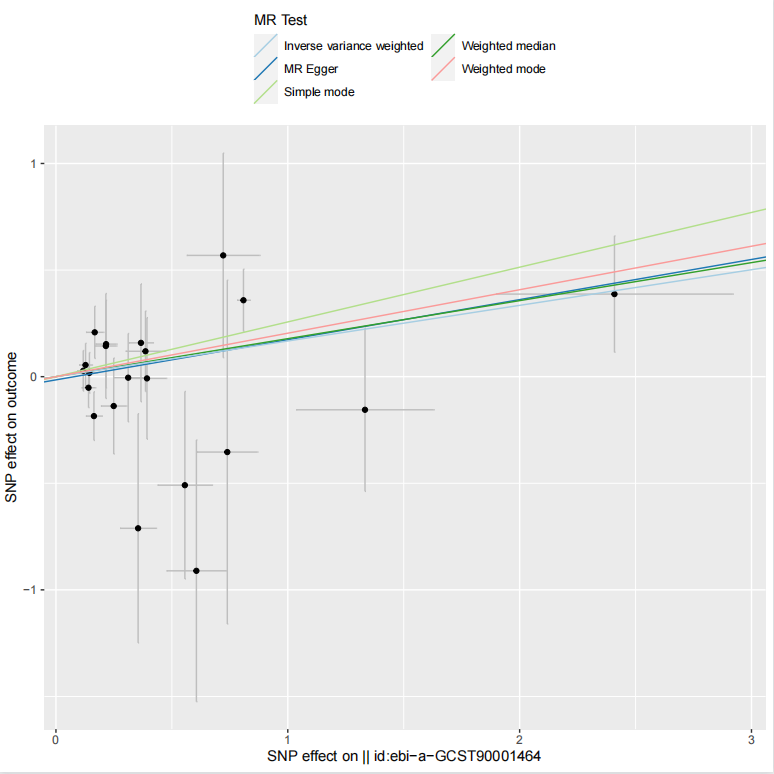


The leave-one-out, funnel, and scatter plots of the causal effect of CD86+ myeloid DC AC on polymyositis.


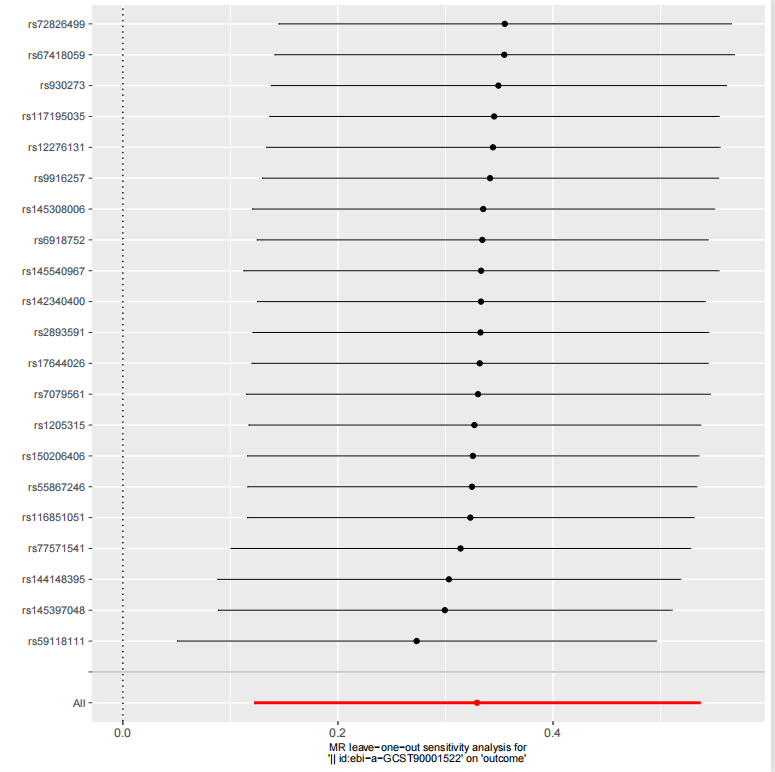


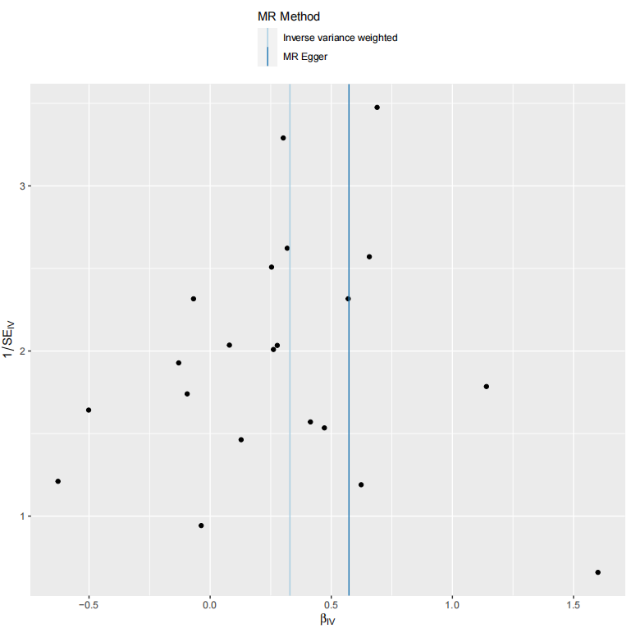

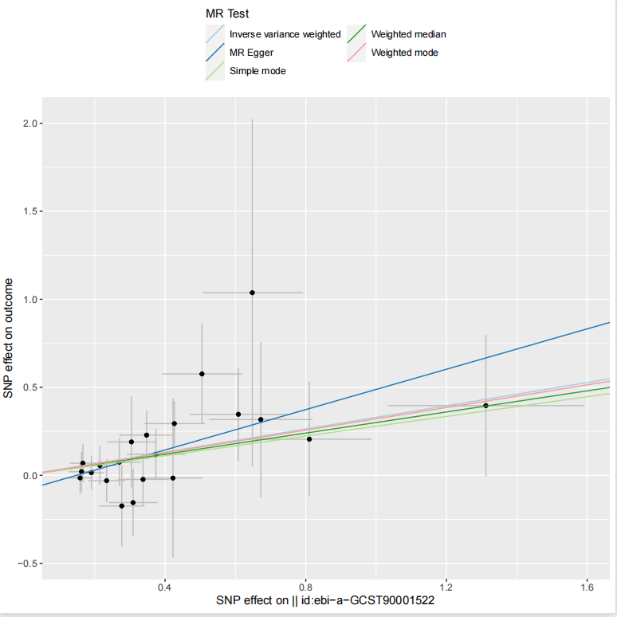


The leave-one-out, funnel, and scatter plots of the causal effect of CD33- HLA DR- AC on polymyositis.


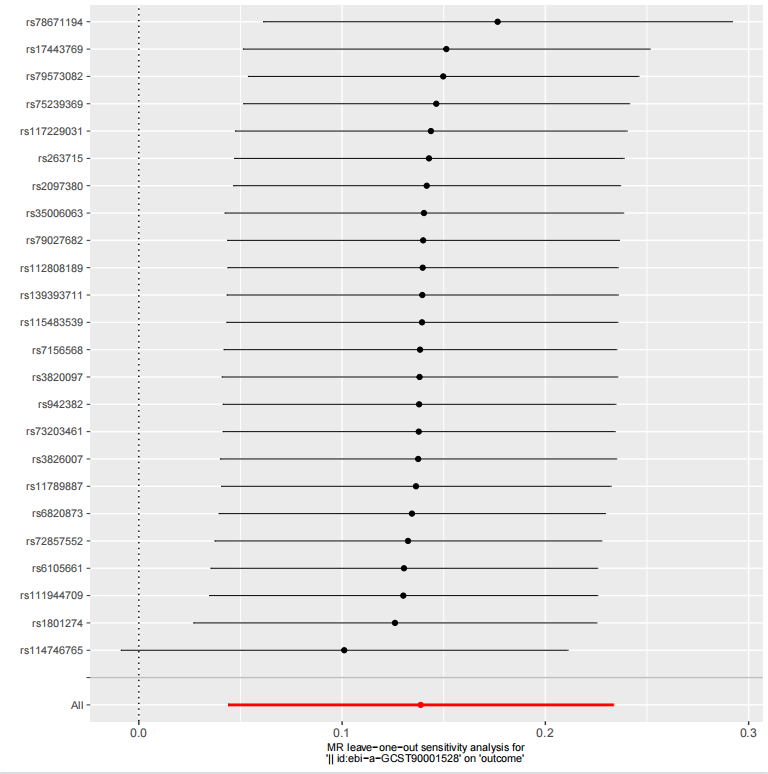


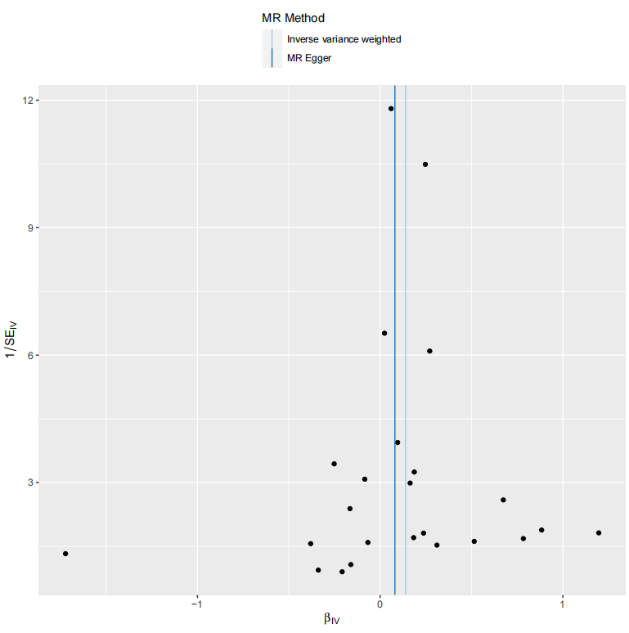

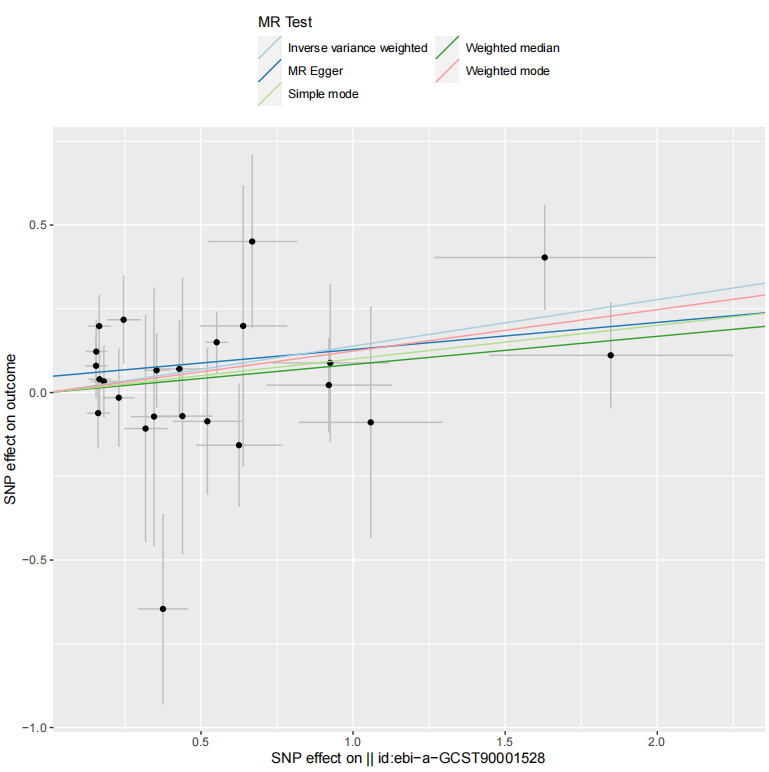


The leave-one-out, funnel, and scatter plots of the causal effect of CD33dim HLA DR+ CD11b- %CD33dim HLA DR+ on polymyositis.


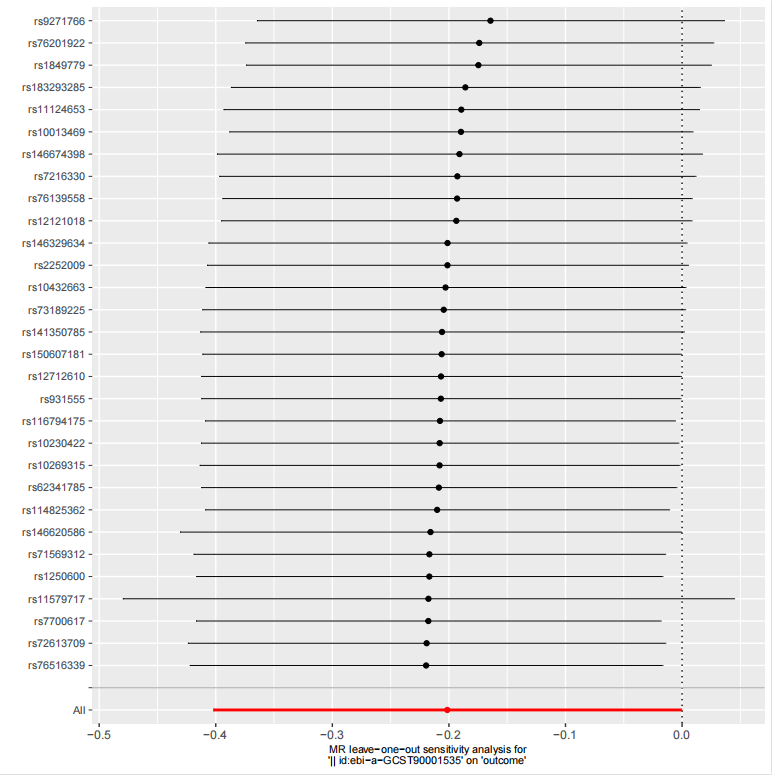


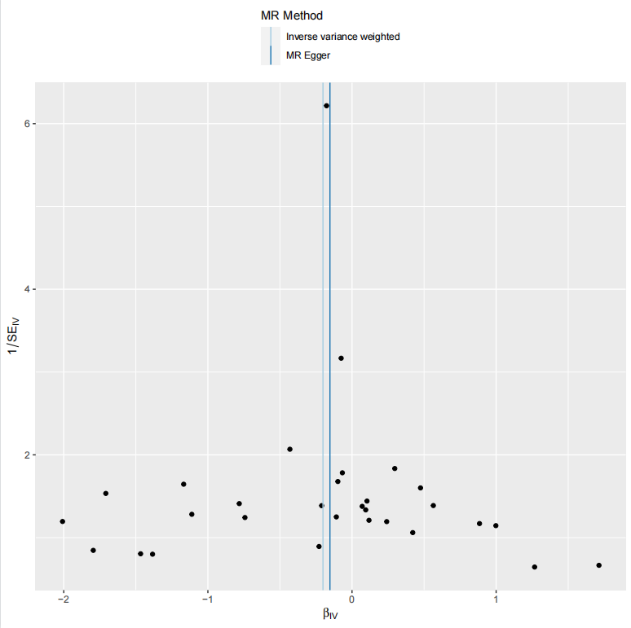

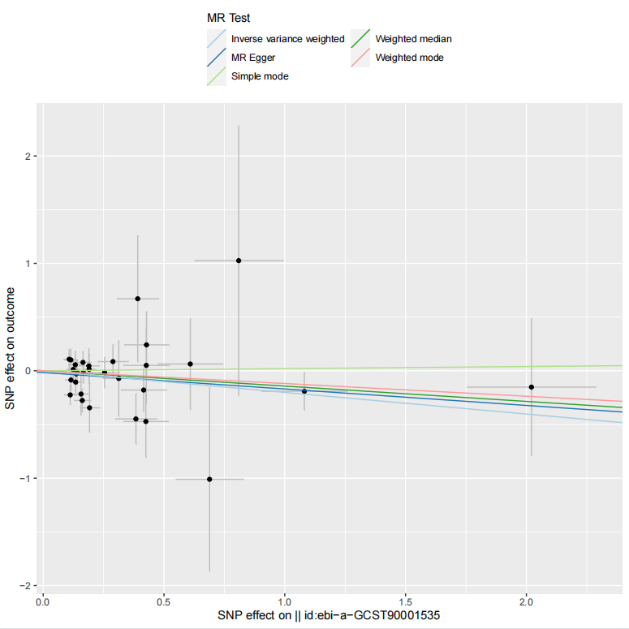


The leave-one-out, funnel, and scatter plots of the causal effect of CD45RA- CD4+ %CD4+ on polymyositis.


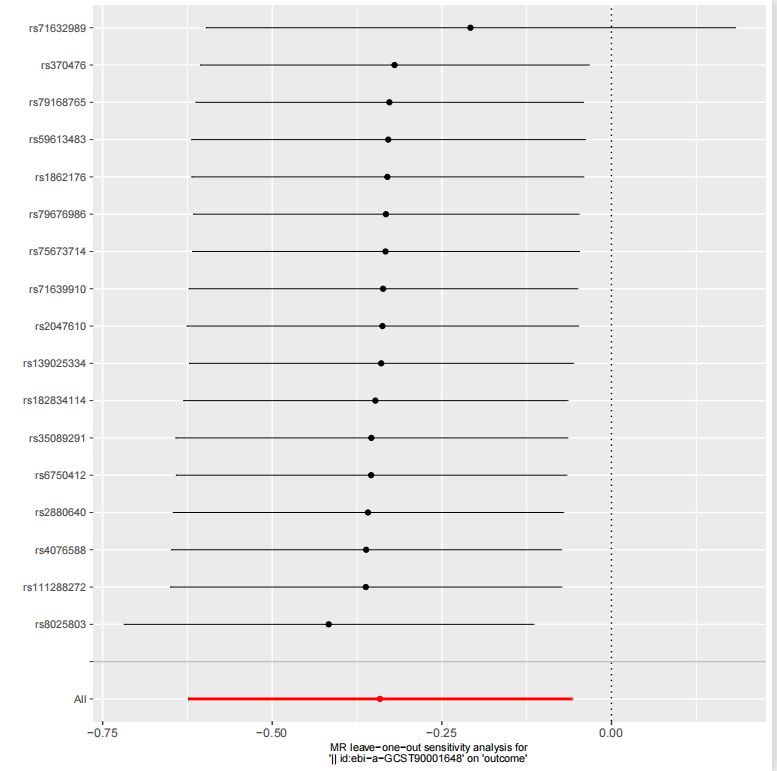


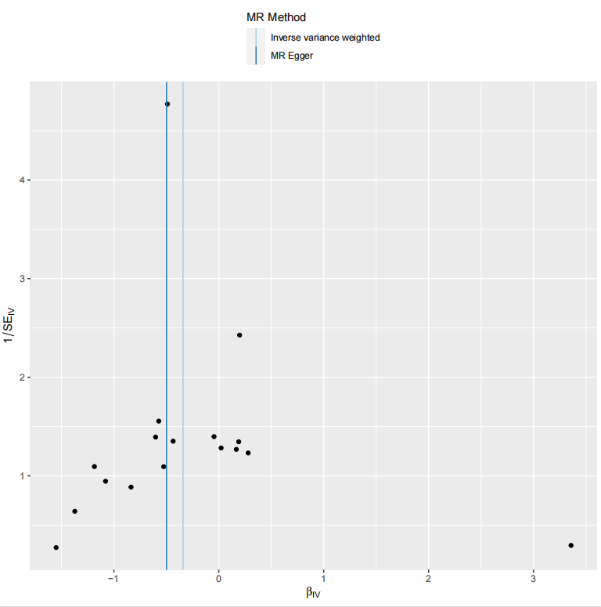

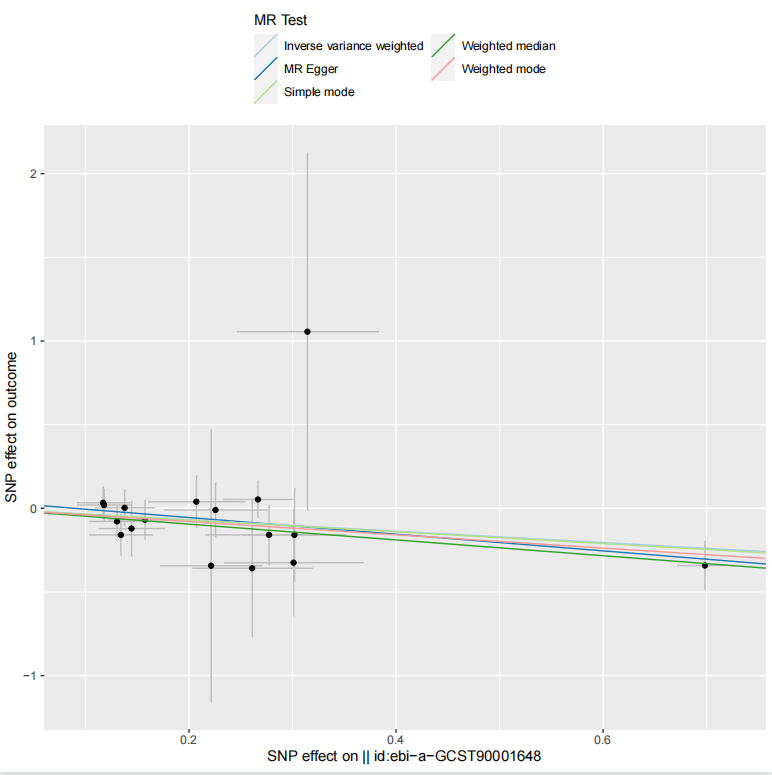


The leave-one-out, funnel, and scatter plots of the causal effect of HLA DR+ NK AC on polymyositis.


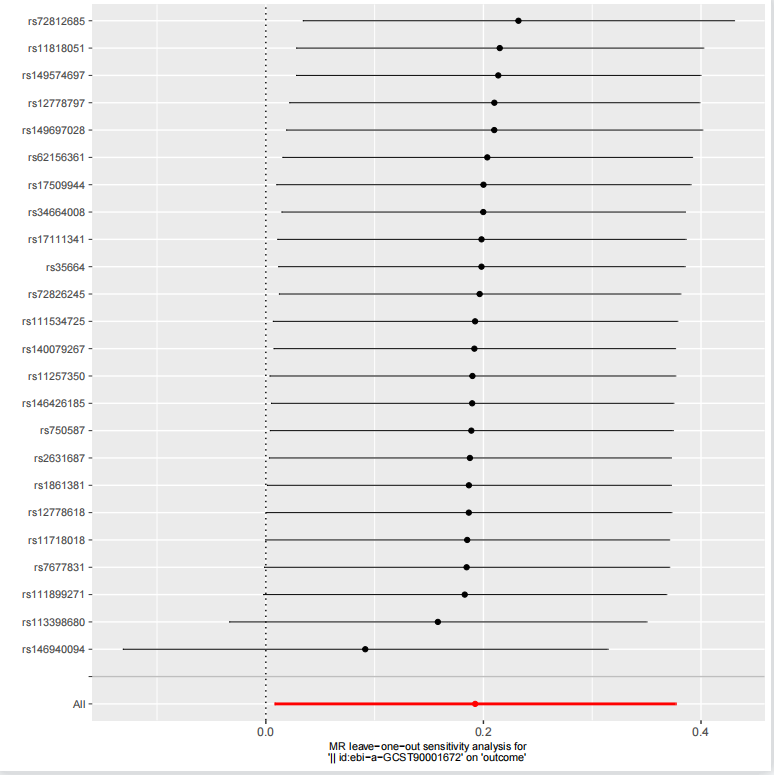


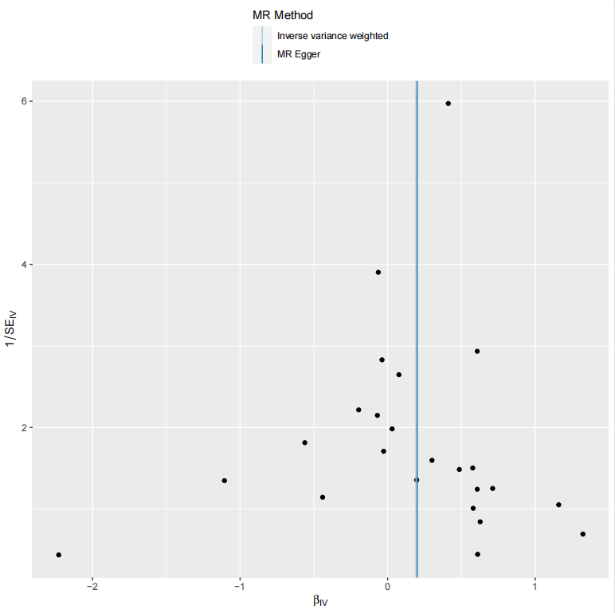

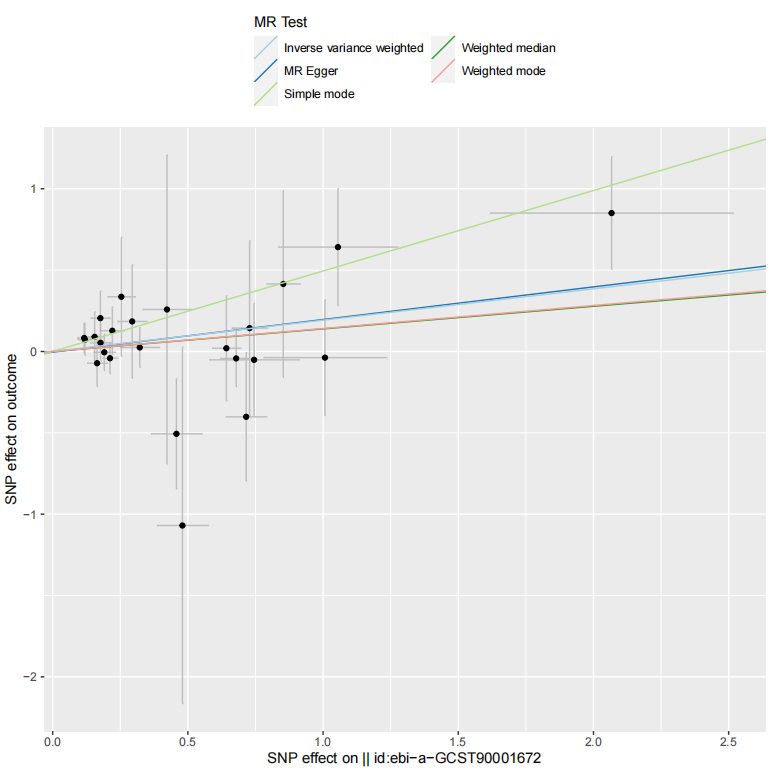


The leave-one-out, funnel, and scatter plots of the causal effect of CD39+ CD8br AC on polymyositis.


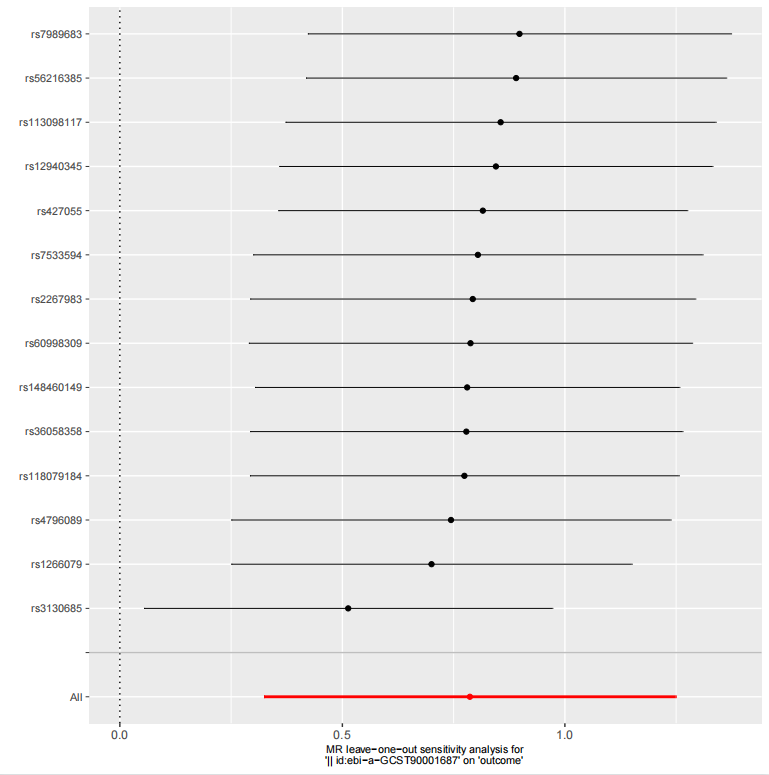


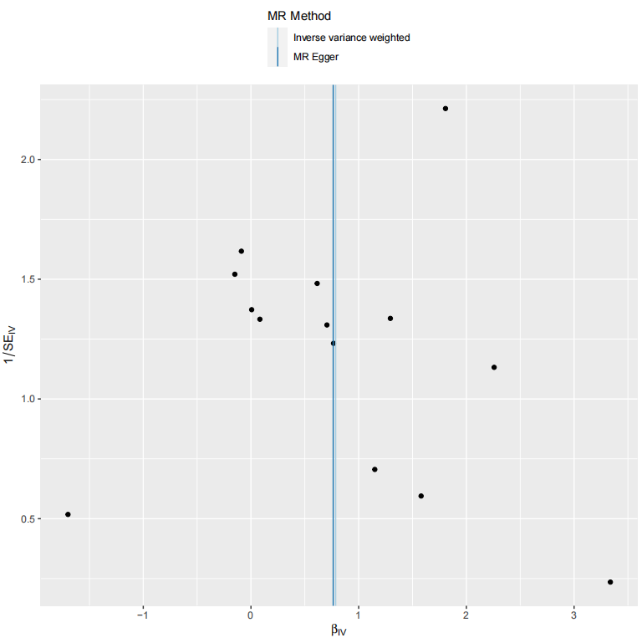

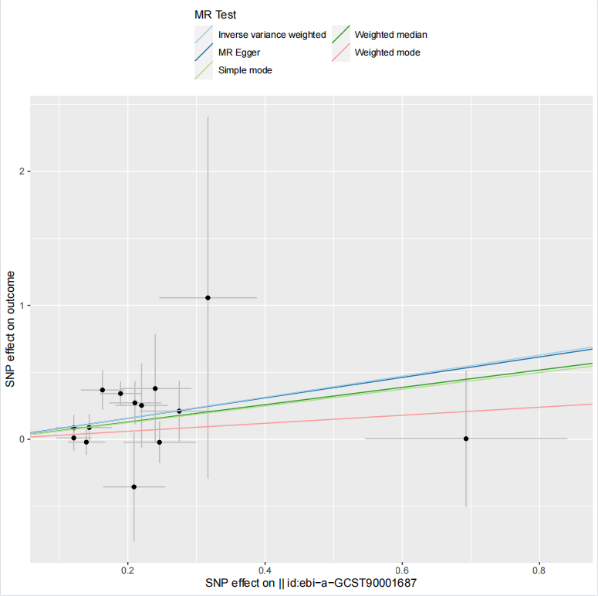


The leave-one-out, funnel, and scatter plots of the causal effect of CD28- CD8br AC on polymyositis.


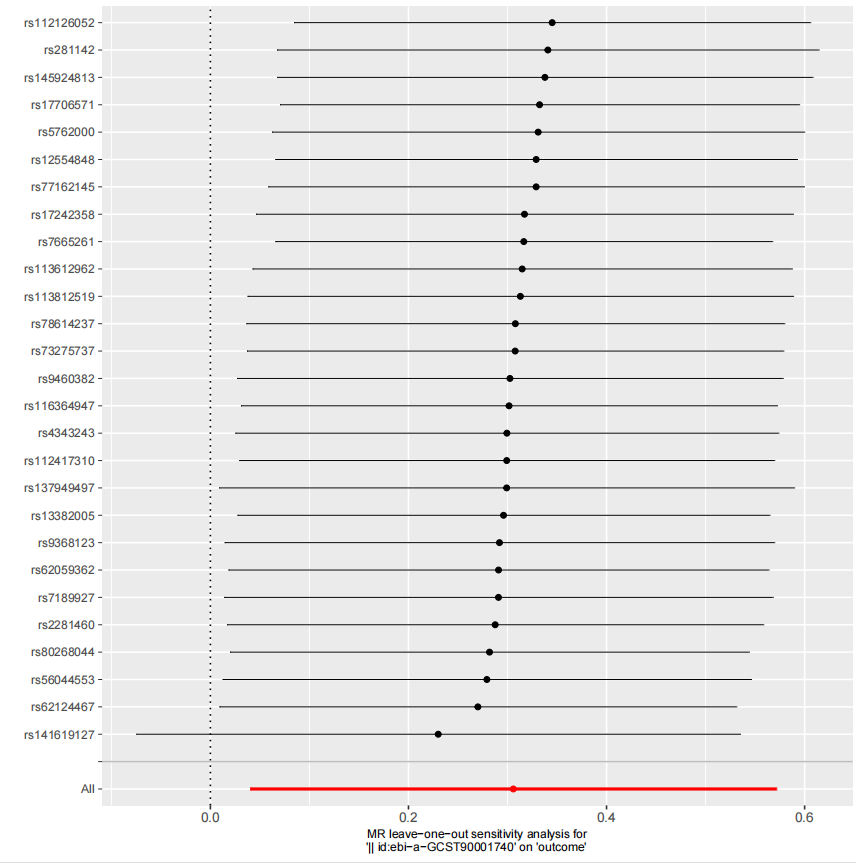


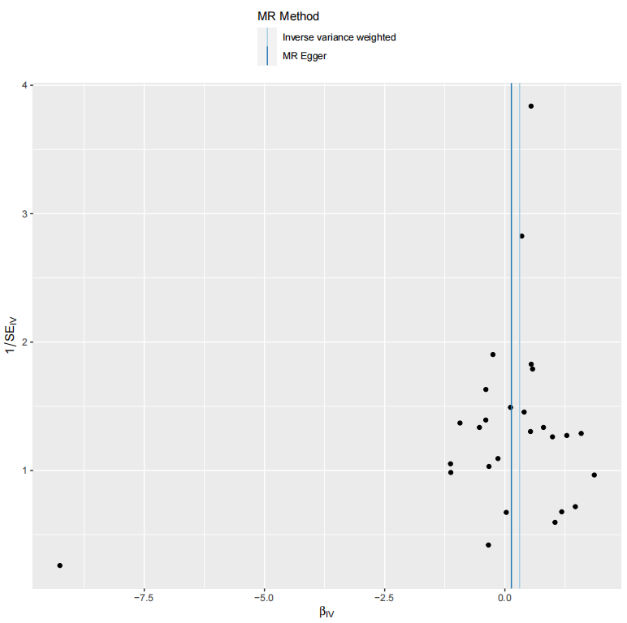

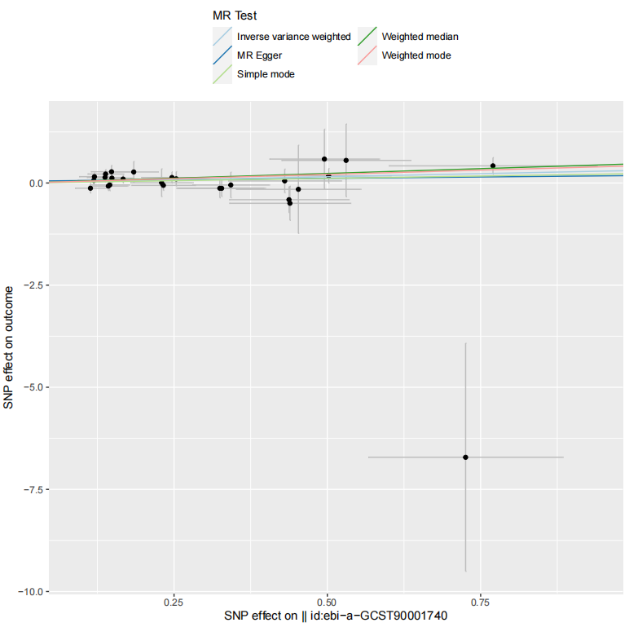


The leave-one-out, funnel, and scatter plots of the causal effect of CD19 on sw mem on polymyositis.


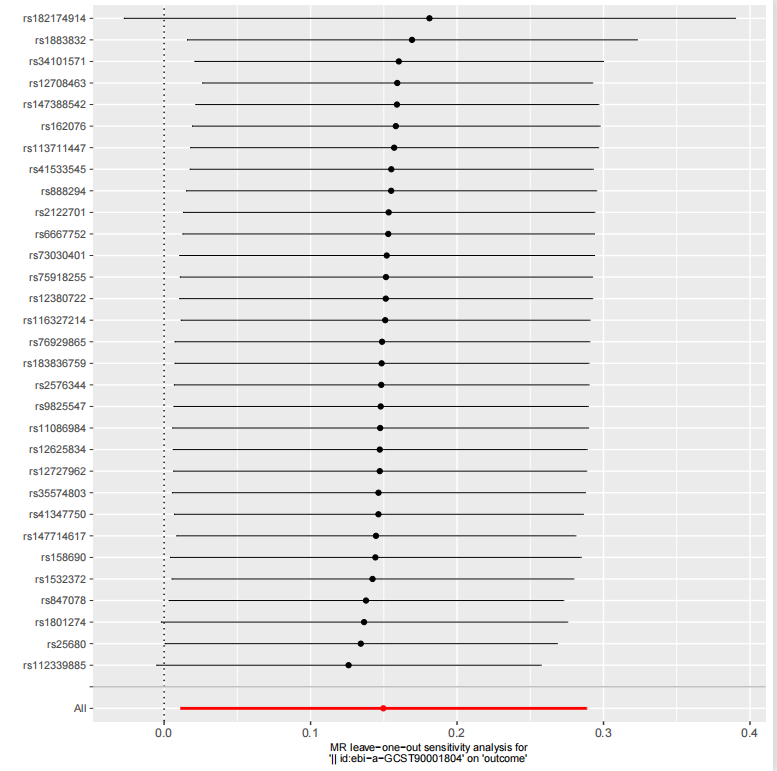


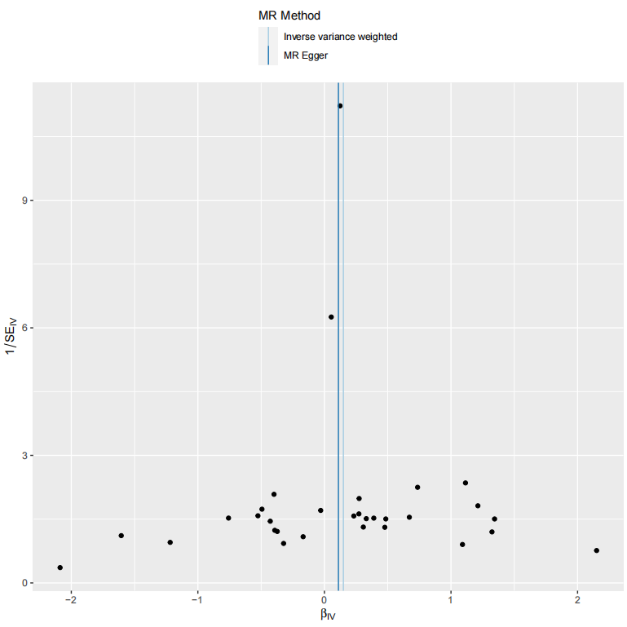

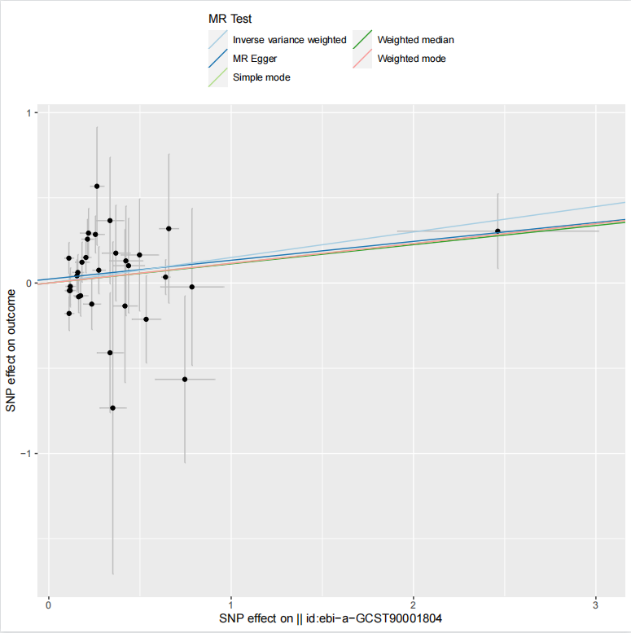


The leave-one-out, funnel, and scatter plots of the causal effect of CD27 on IgD- CD38dim on polymyositis.


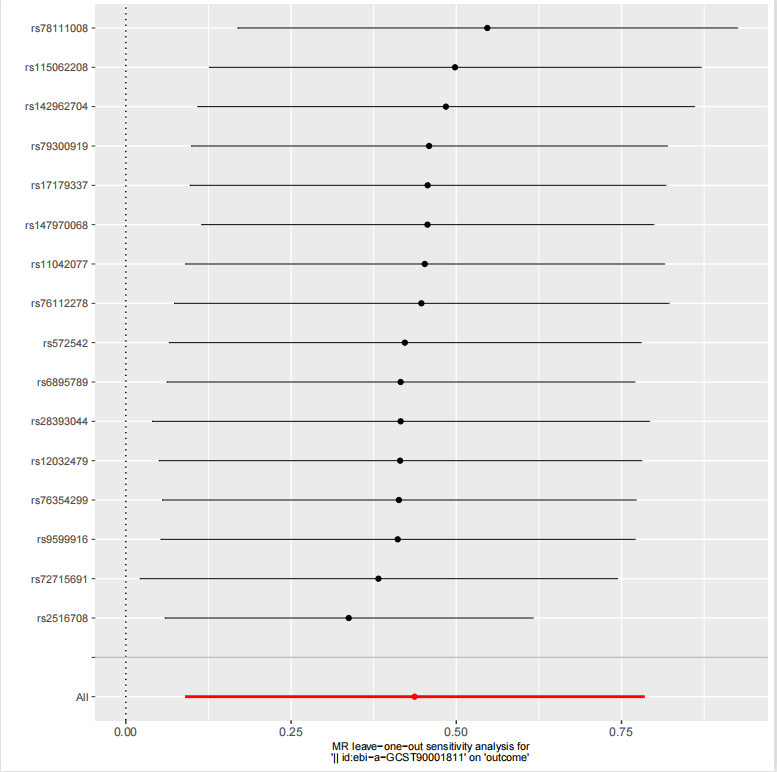


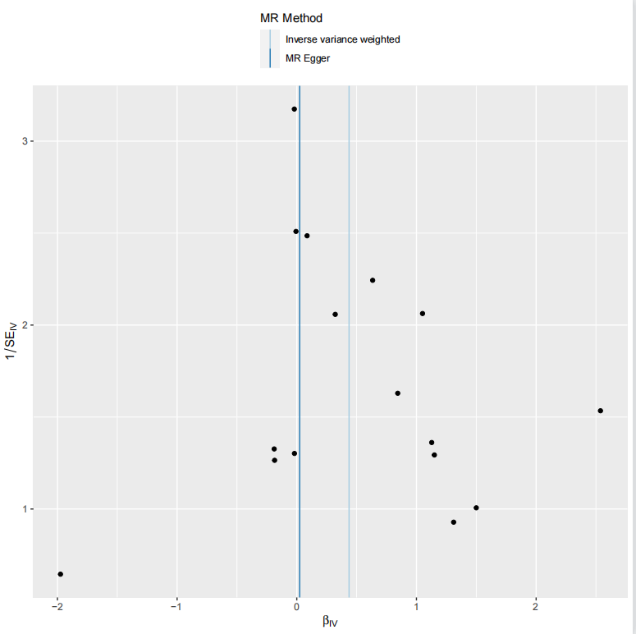

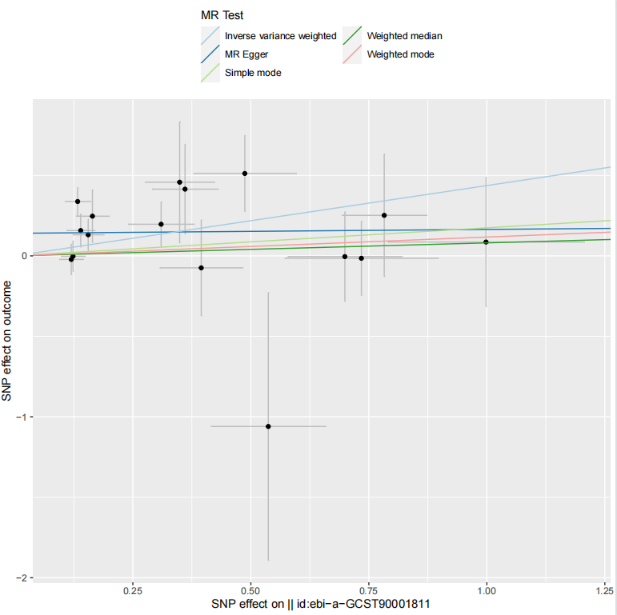


The leave-one-out, funnel, and scatter plots of the causal effect of CD38 on IgD+ CD24-on polymyositis.


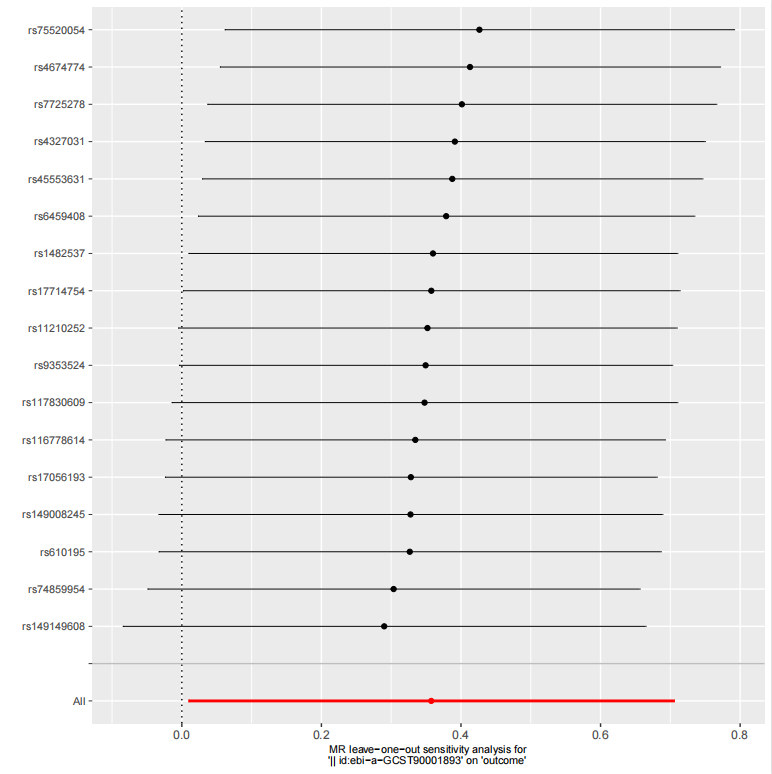


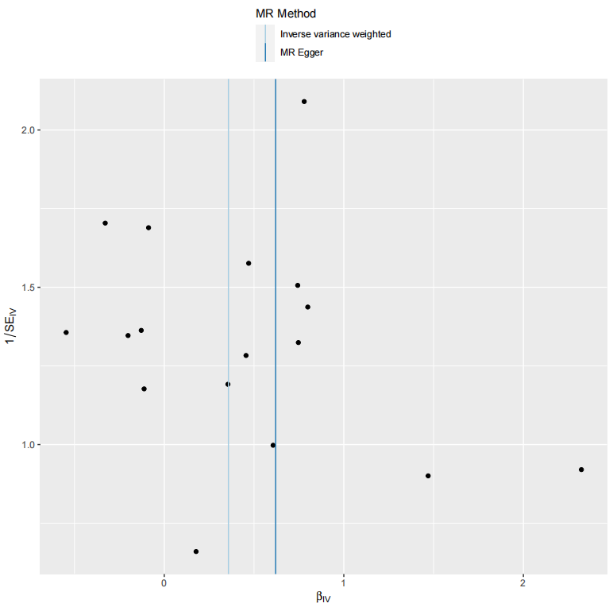

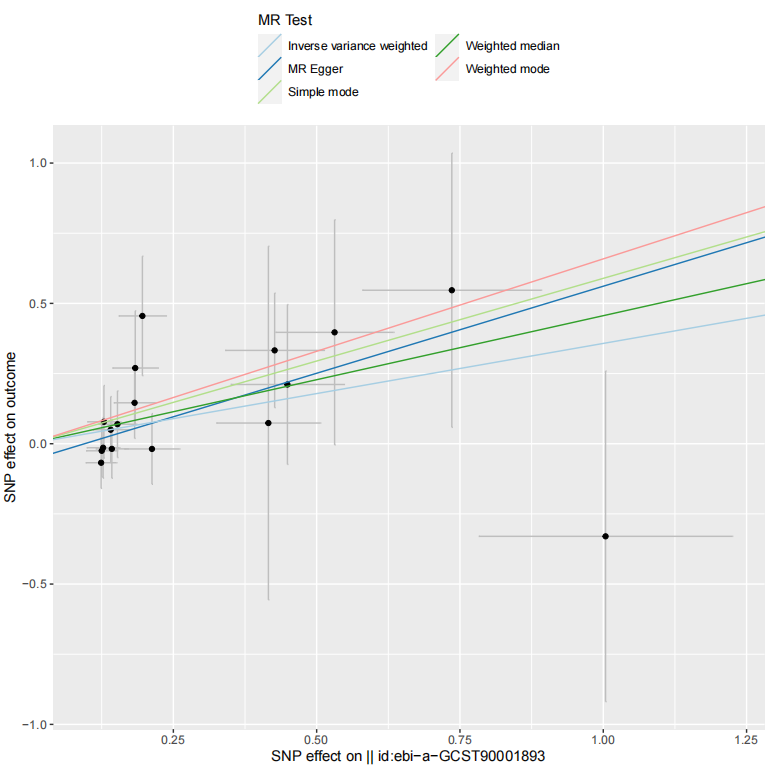


The leave-one-out, funnel, and scatter plots of the causal effect of CD28 on CD28+ CD45RA- CD8br on polymyositis.


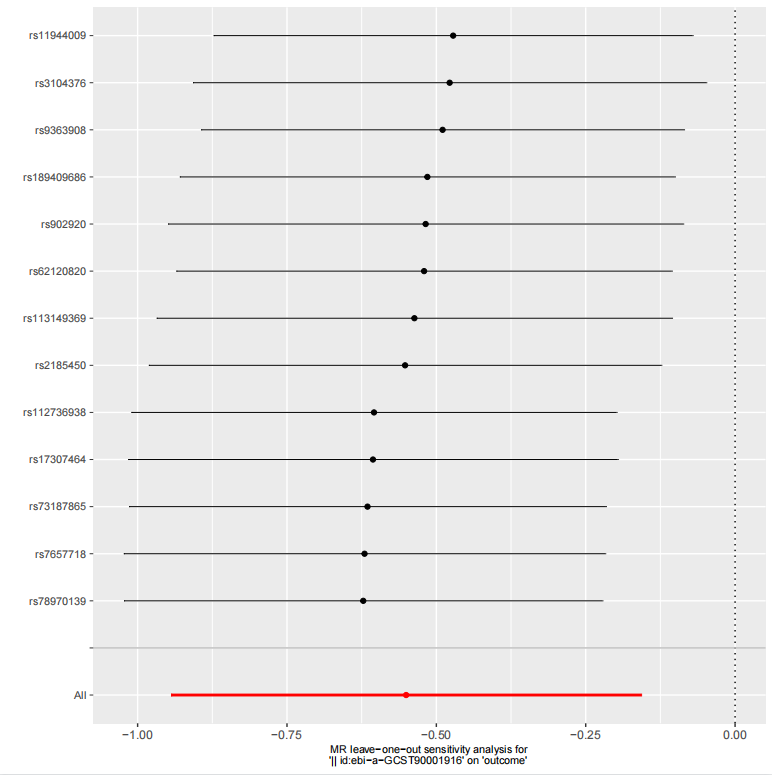


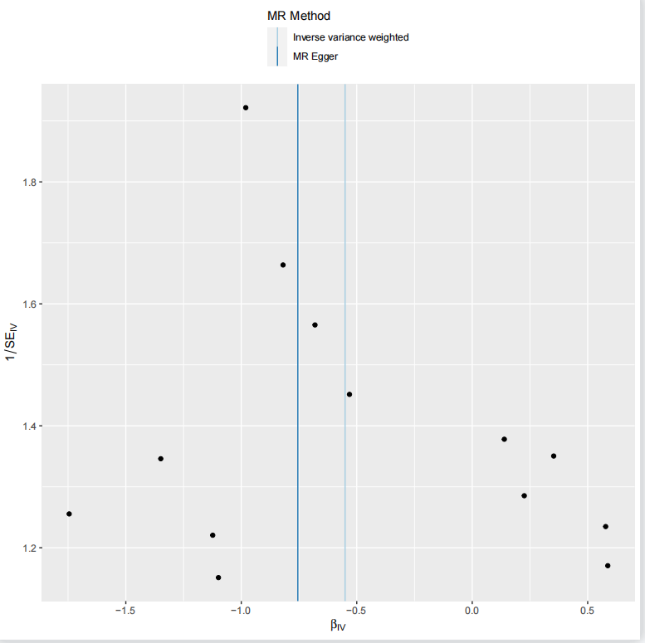

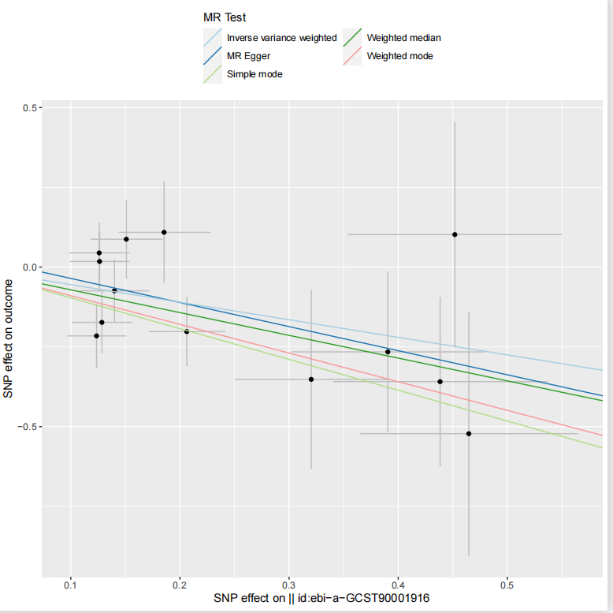


The leave-one-out, funnel, and scatter plots of the causal effect of CD45 on CD4+ on polymyositis.


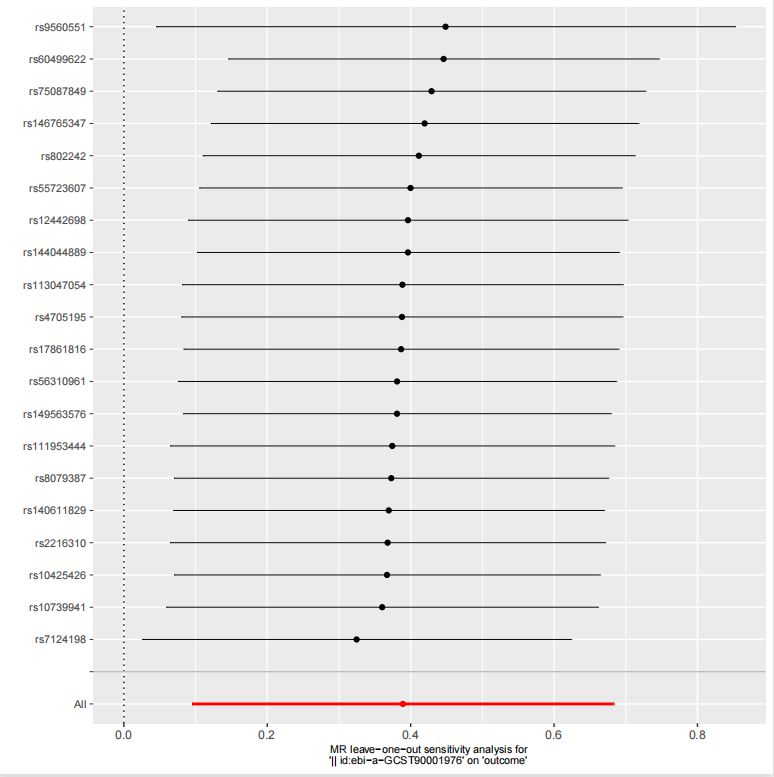


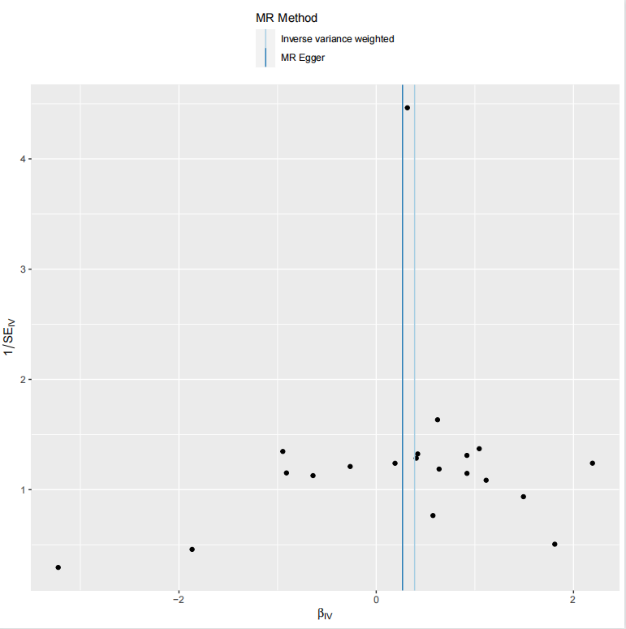

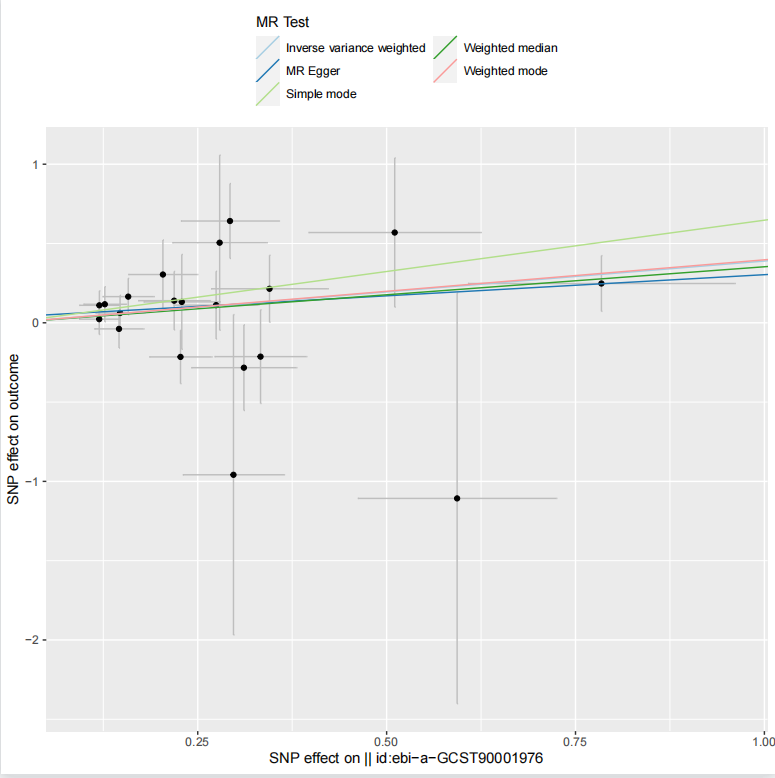


The leave-one-out, funnel, and scatter plots of the causal effect of FSC-A on NKT on polymyositis.


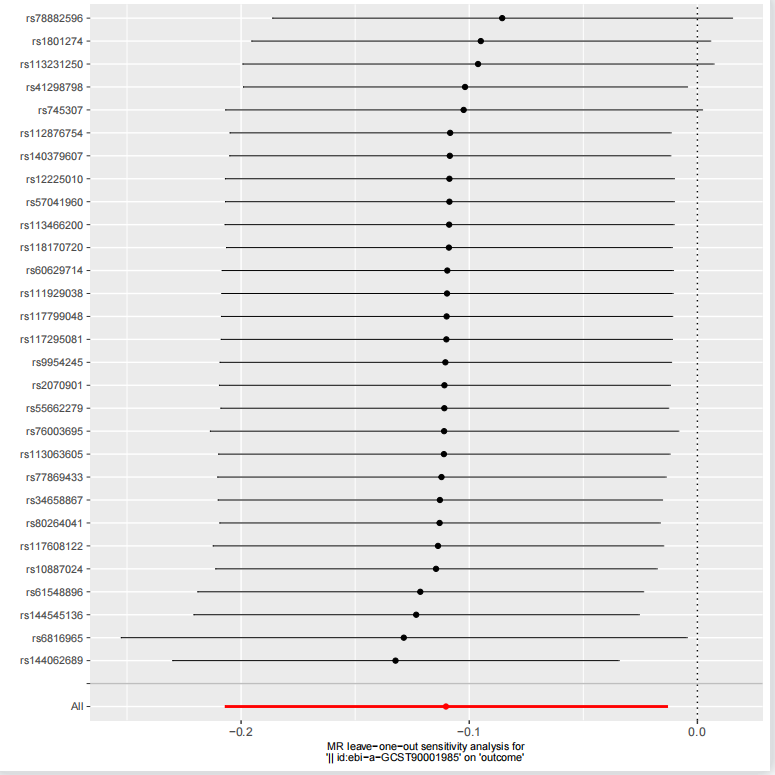


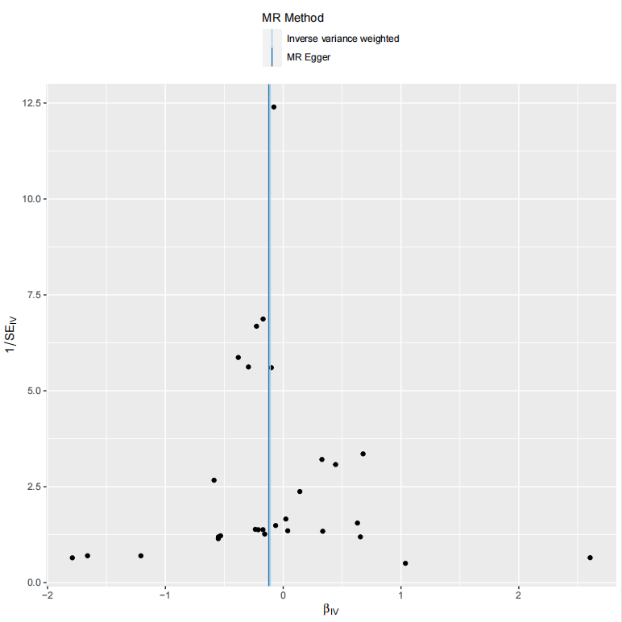

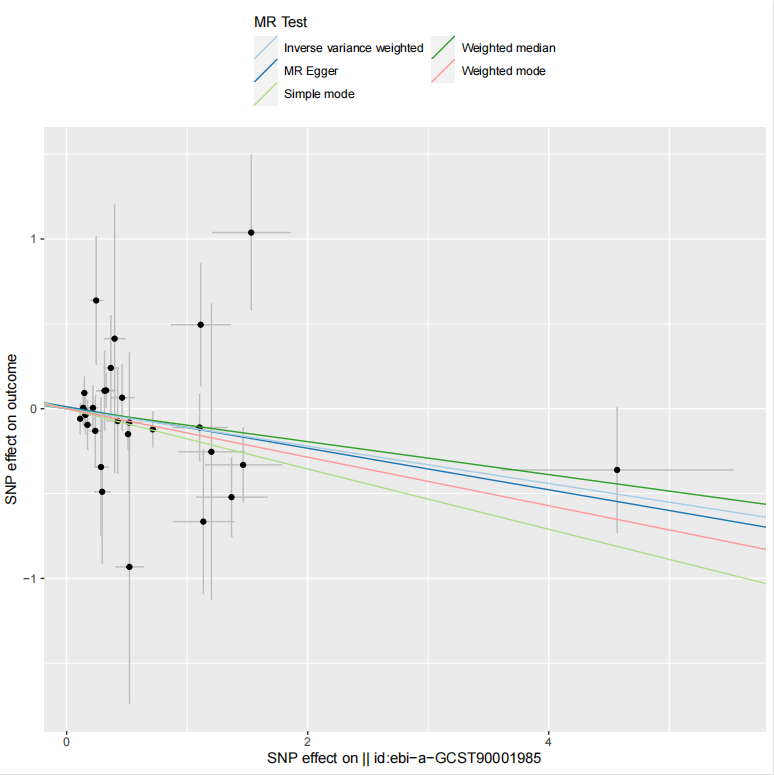


The leave-one-out, funnel, and scatter plots of the causal effect of CD40 on monocytes on polymyositis


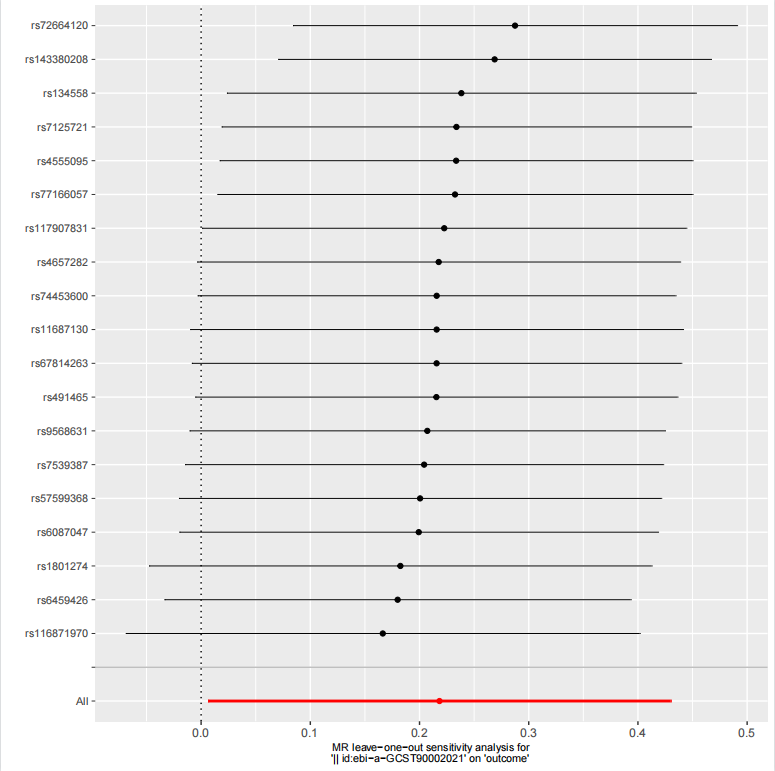


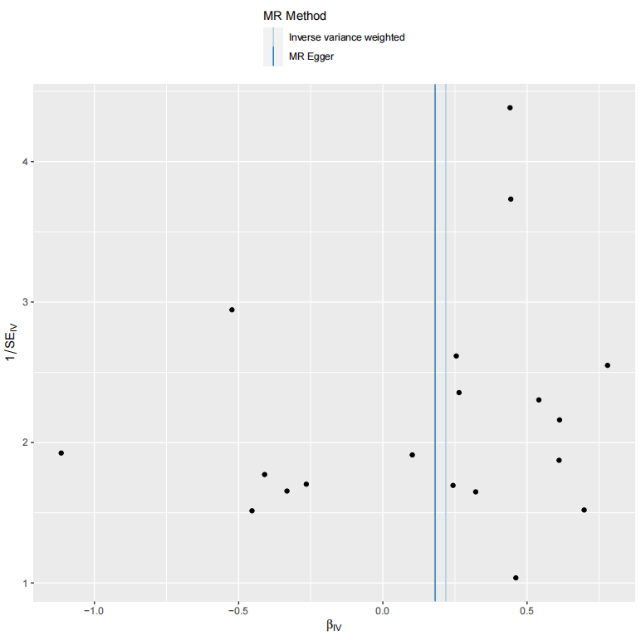

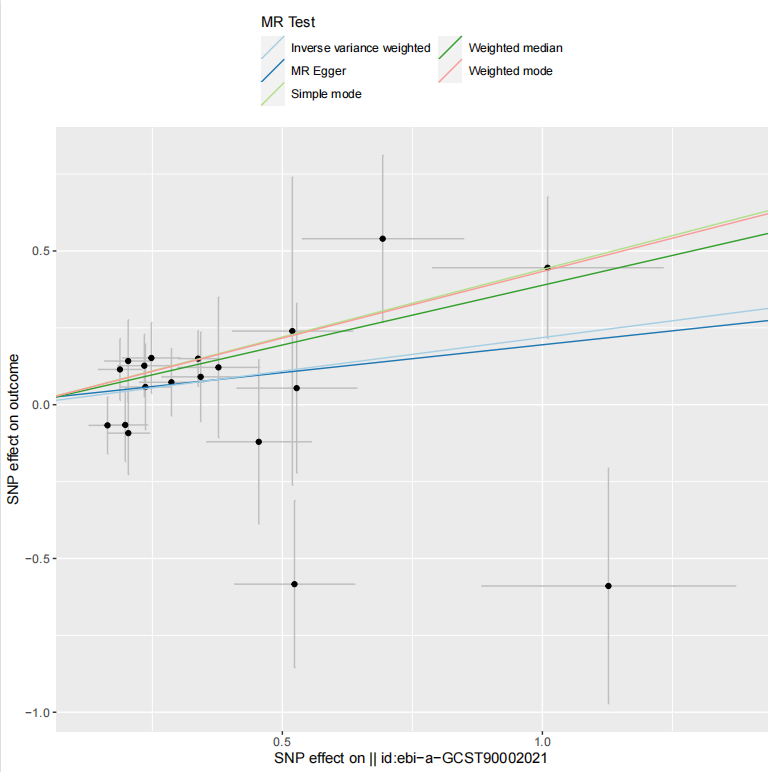


The leave-one-out, funnel, and scatter plots of the causal effect of CD14 on CD33dim HLA DR+ CD11b+ on polymyositis


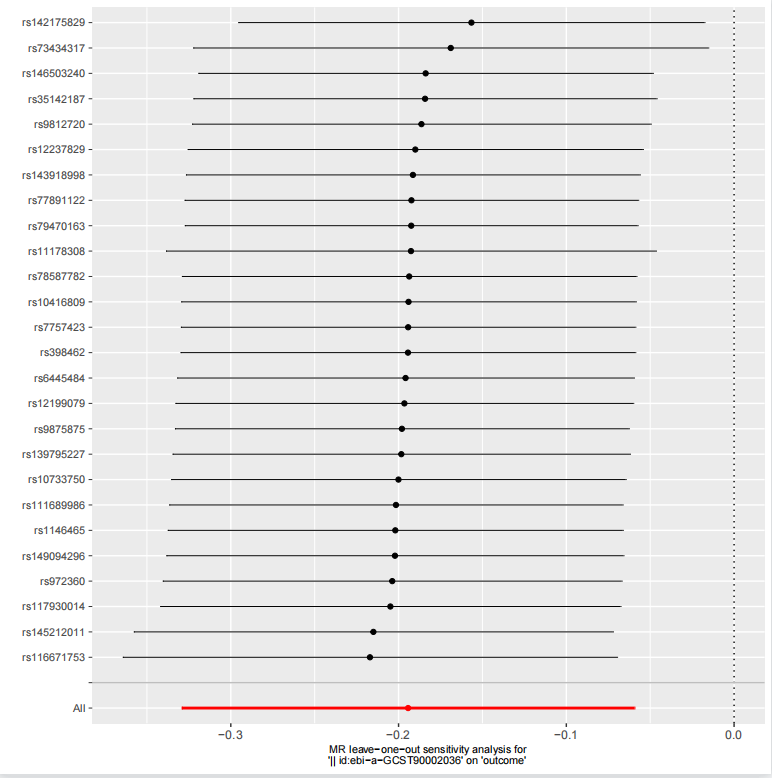


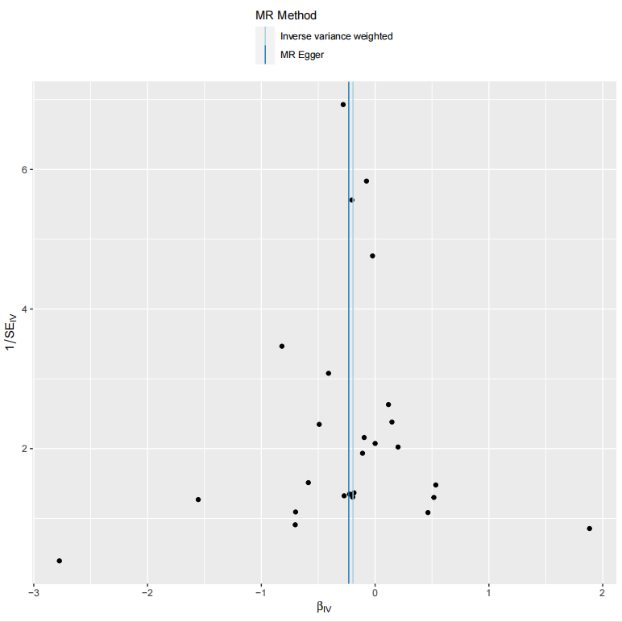

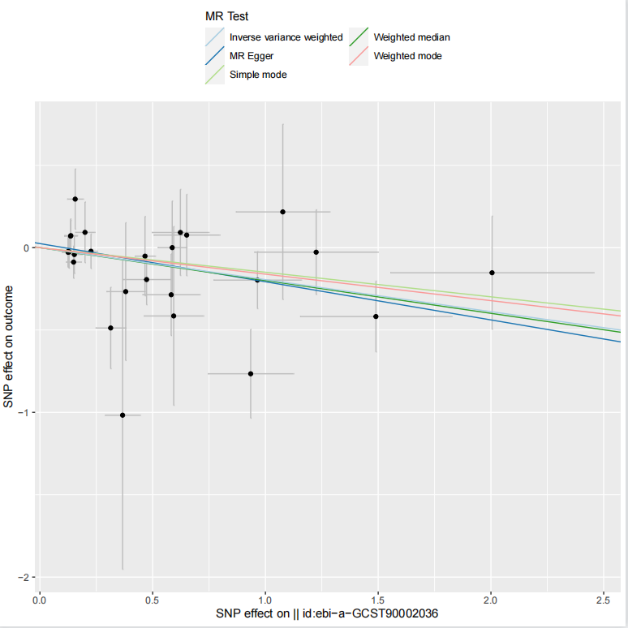


The leave-one-out, funnel, and scatter plots of the causal effect of CD80 on CD62L+ myeloid DC on polymyositis.


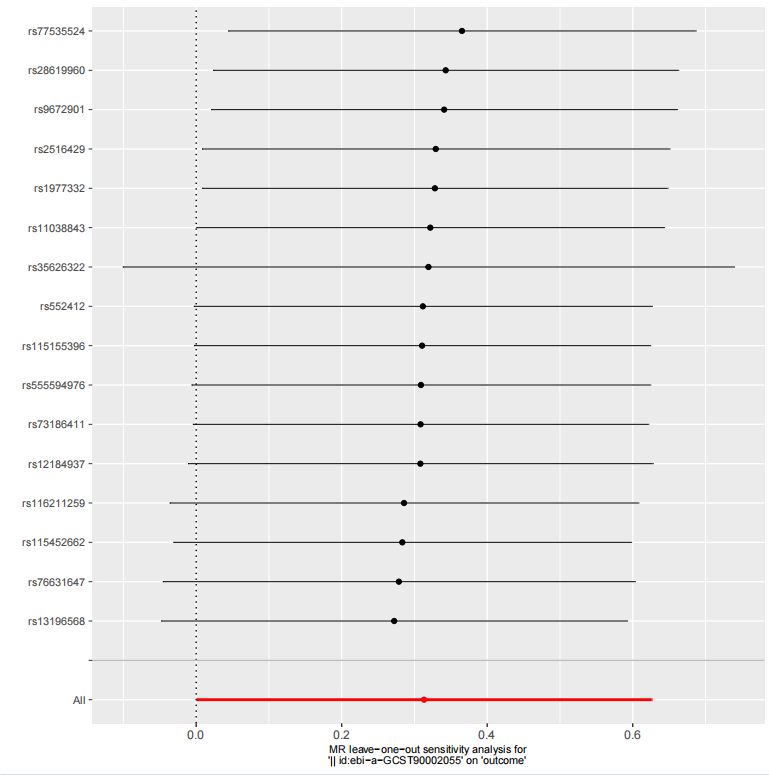

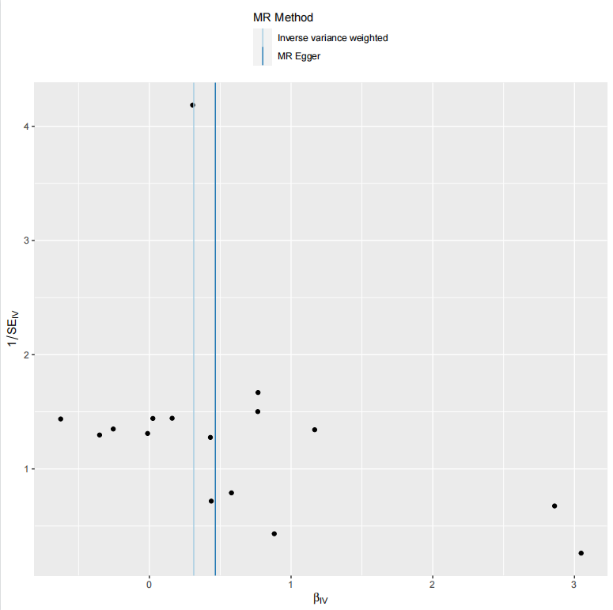

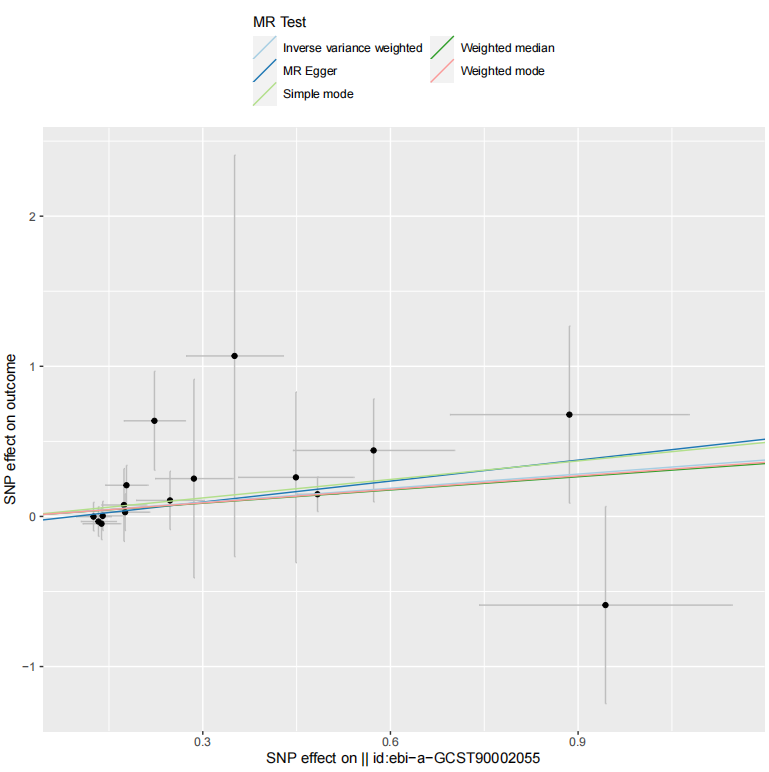


The leave-one-out, funnel, and scatter plots of the causal effect of CD8 on naive CD8br on polymyositis.


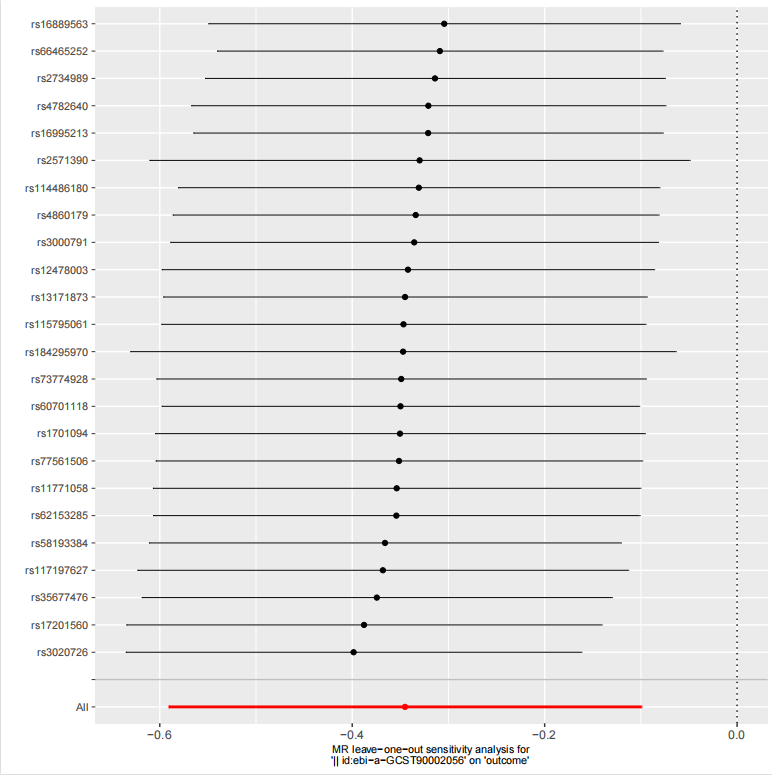


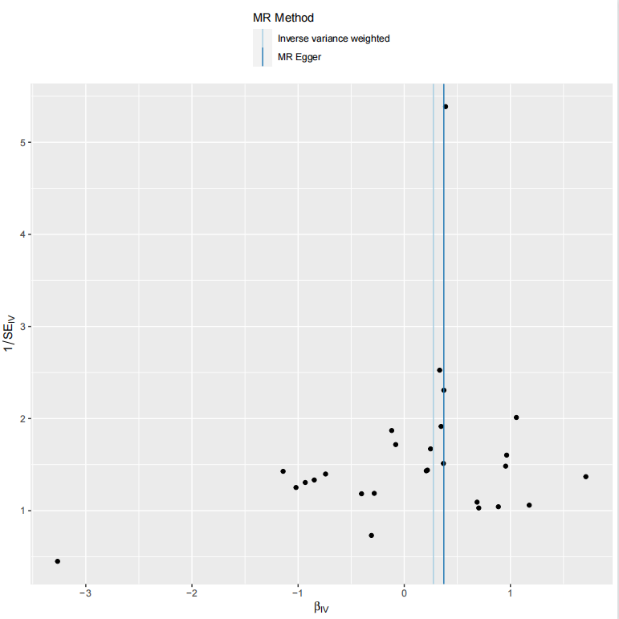

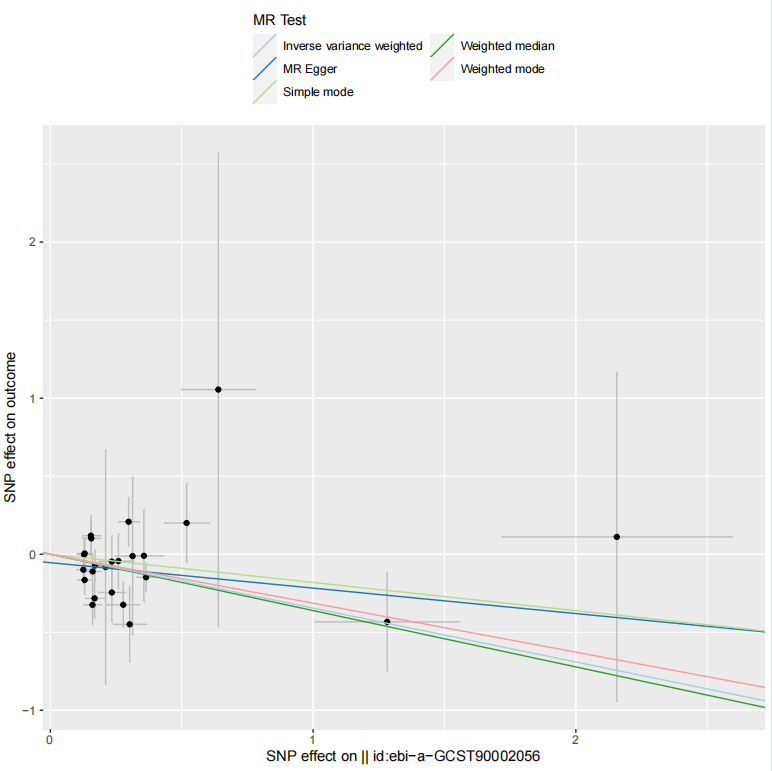


The leave-one-out, funnel, and scatter plots of the causal effect of CD8 on EM CD8br on polymyositis.


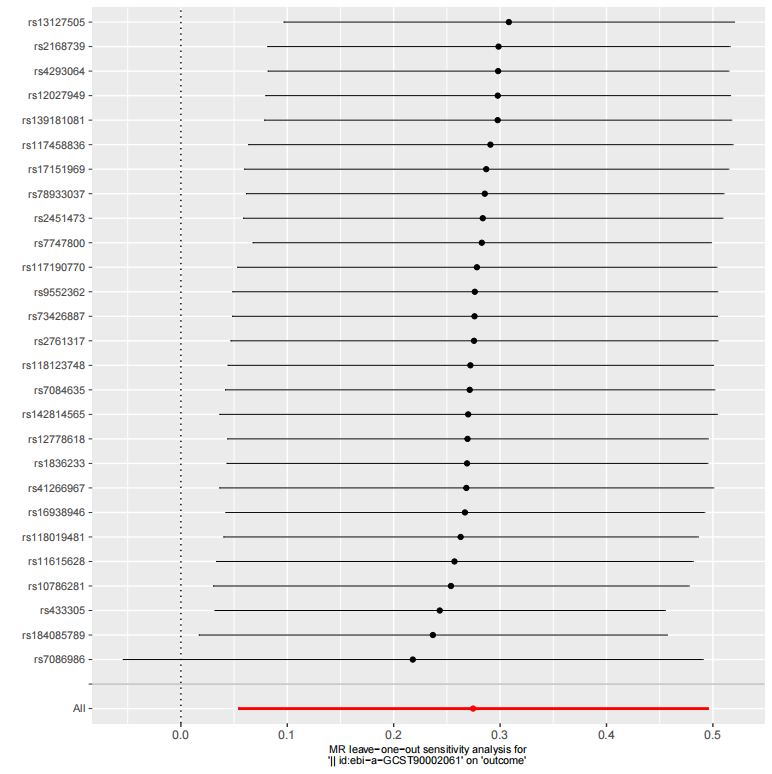

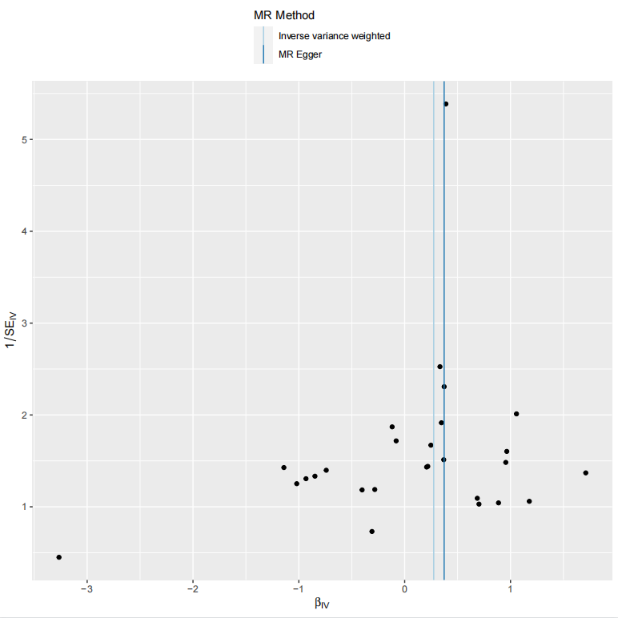

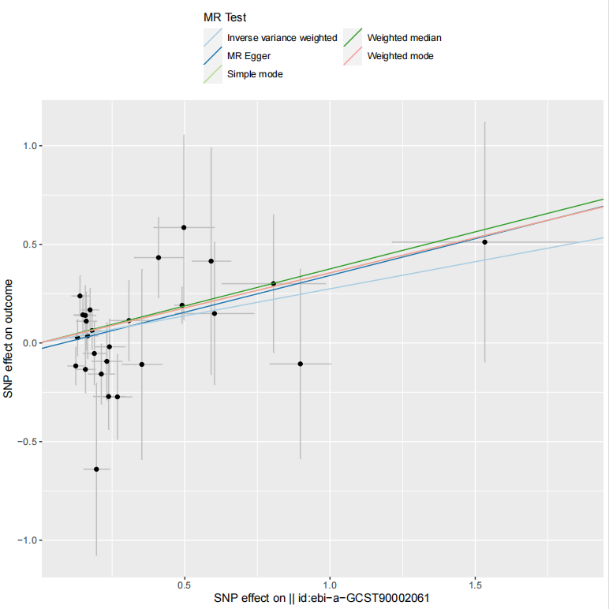


The leave-one-out, funnel, and scatter plots of the causal effect of CD4 on CD39+ CD4+ on polymyositis.


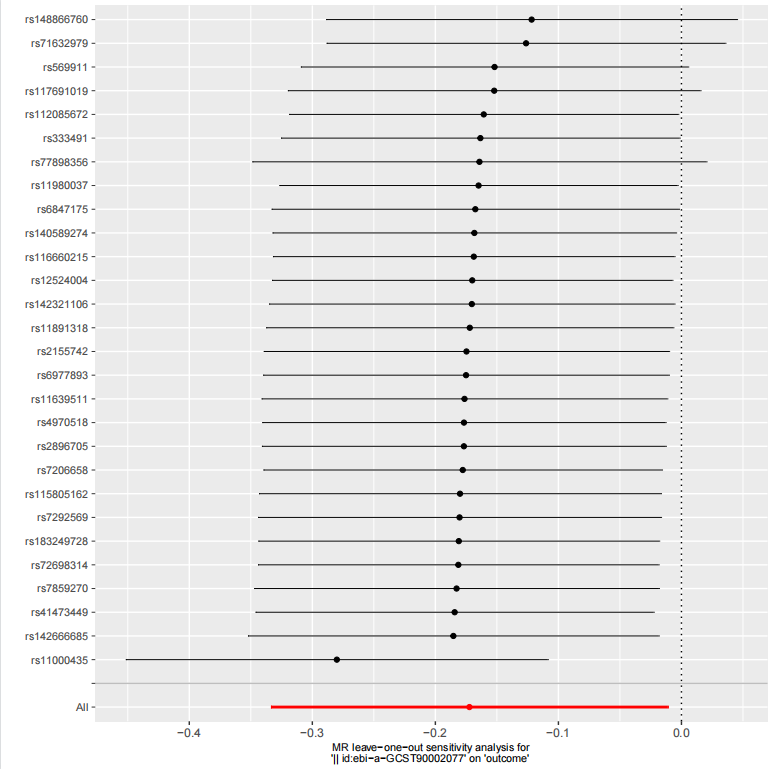

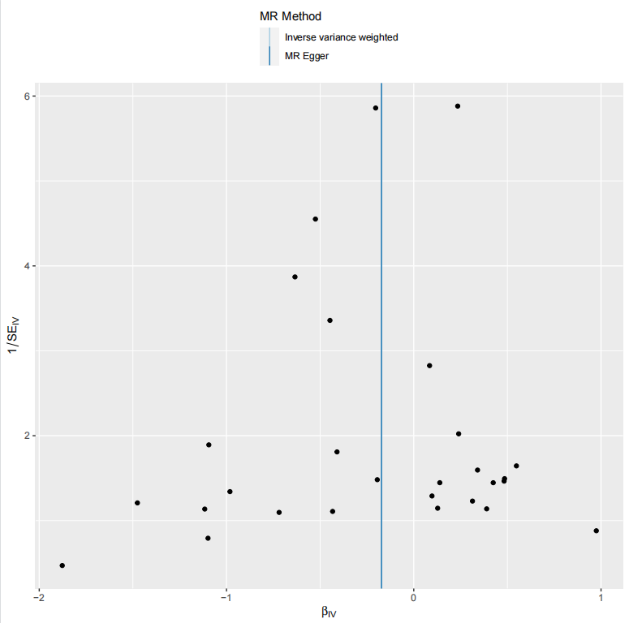

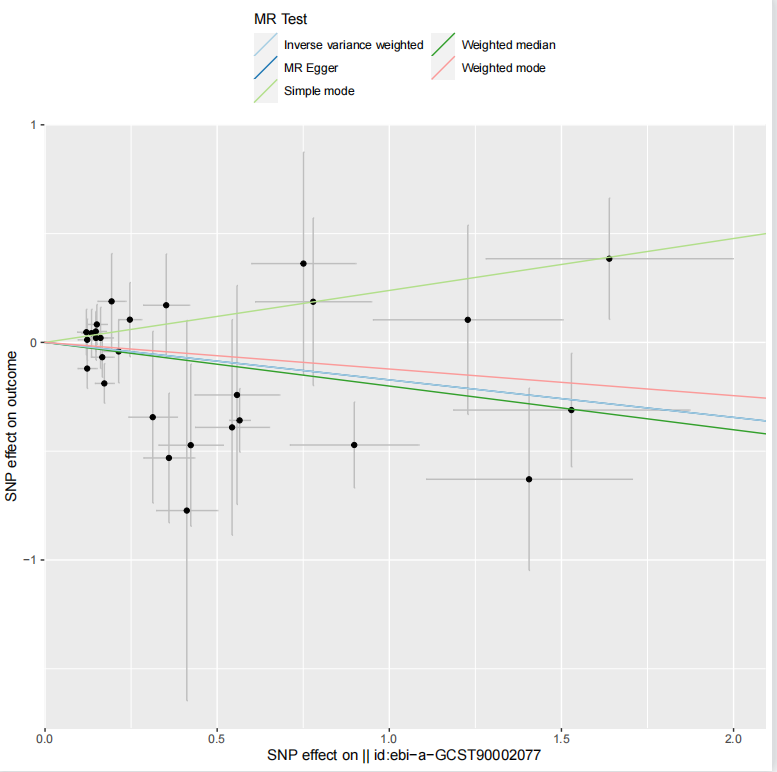


The leave-one-out, funnel, and scatter plots of the causal effect of SSC-A on HLA DR+ NK on polymyositis.


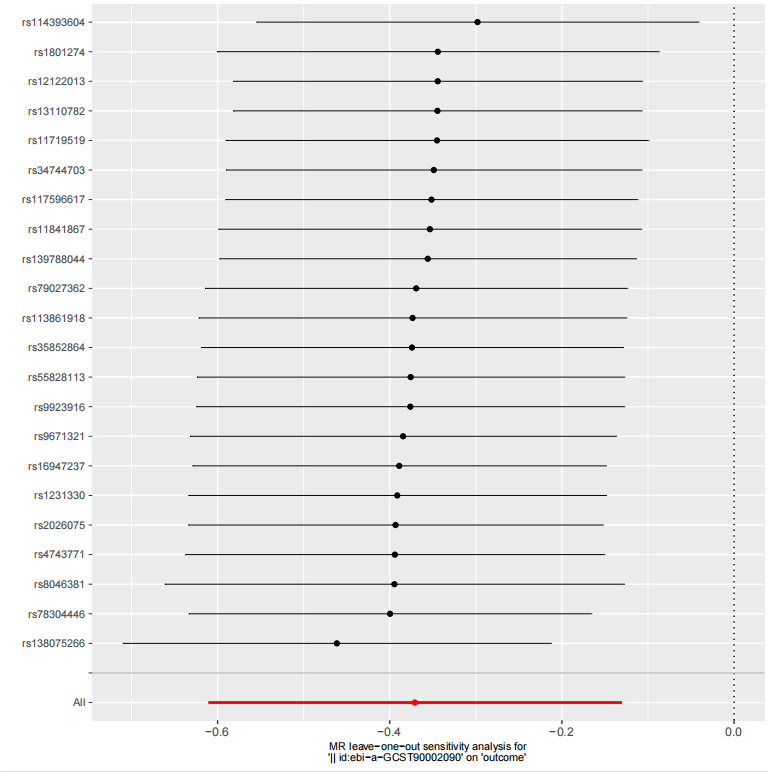


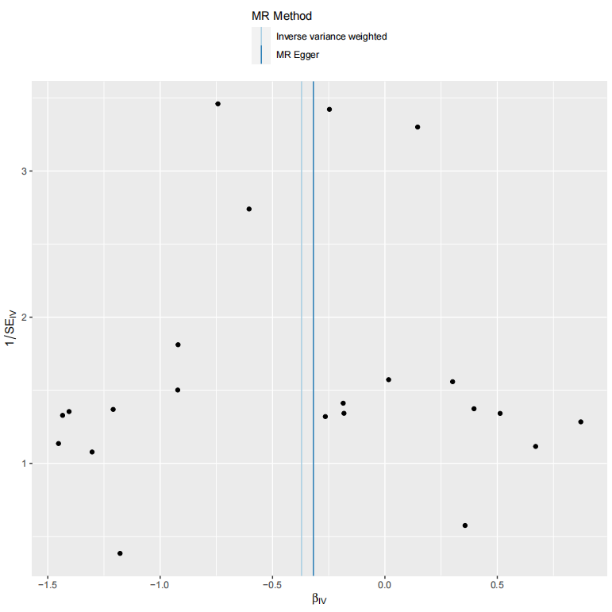

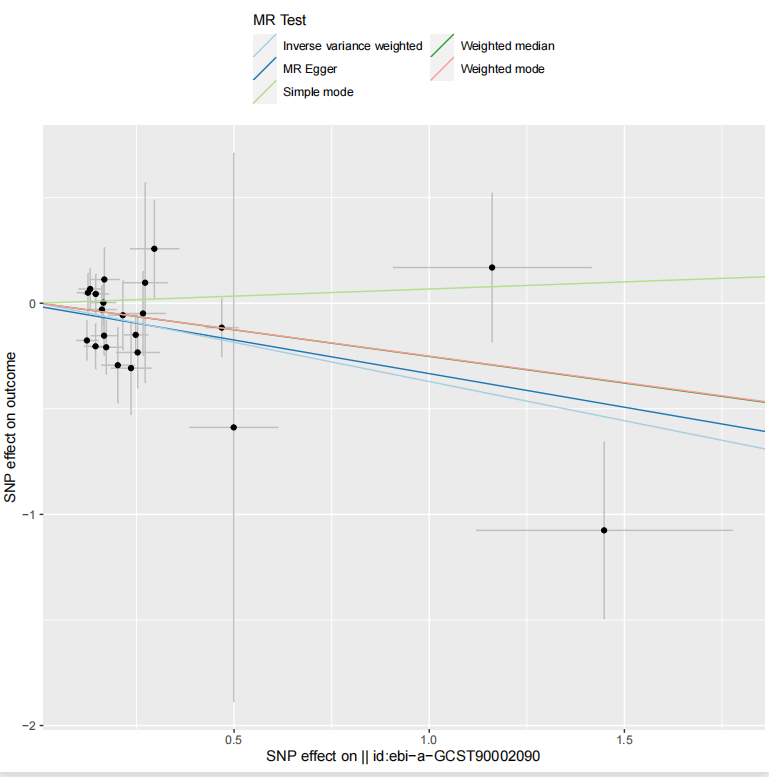


The leave-one-out, funnel, and scatter plots of the causal effect of CD11c on granulocyte on polymyositis.


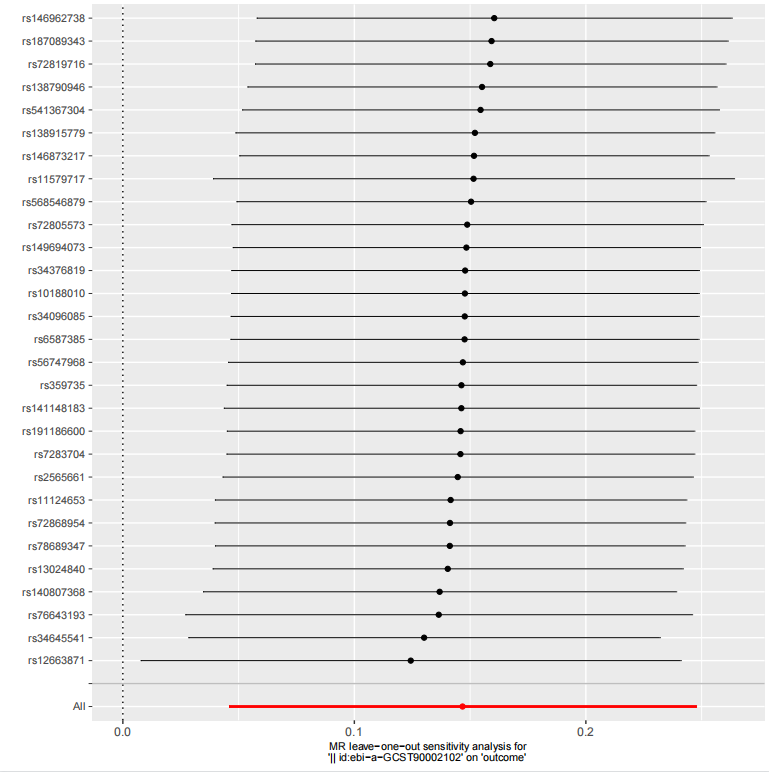


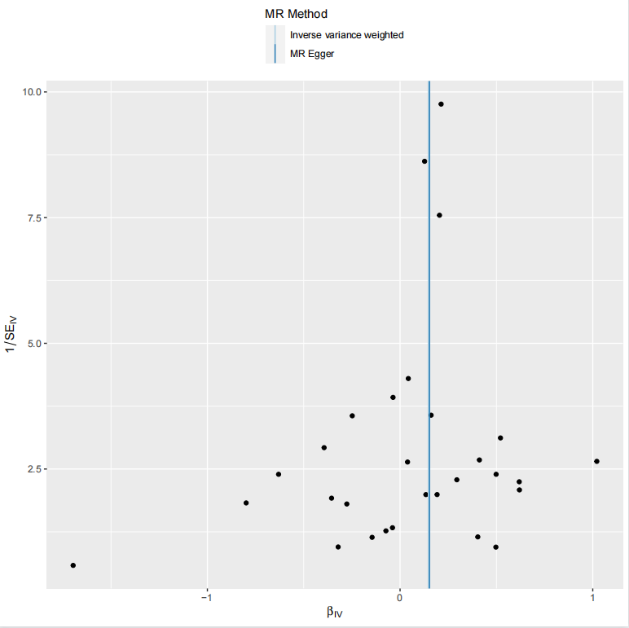

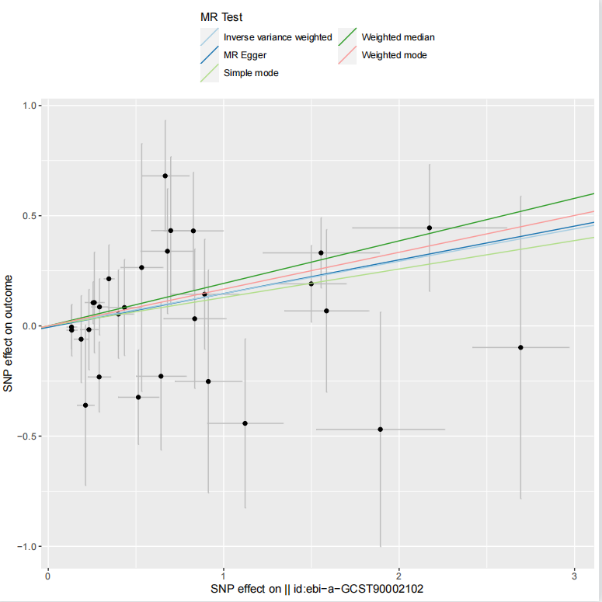


The leave-one-out, funnel, and scatter plots of the causal effect of CD45RA on resting Treg on polymyositis.


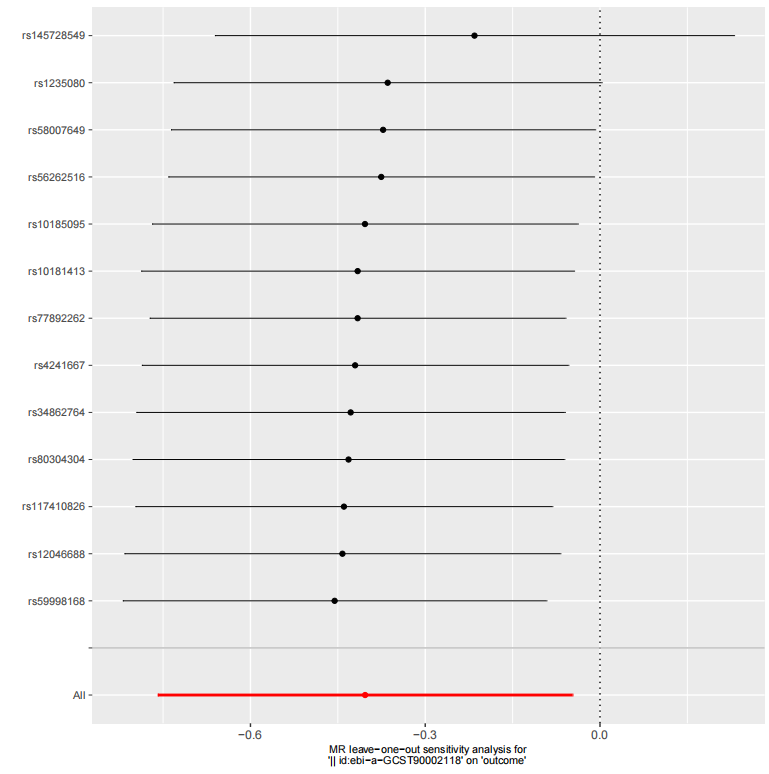

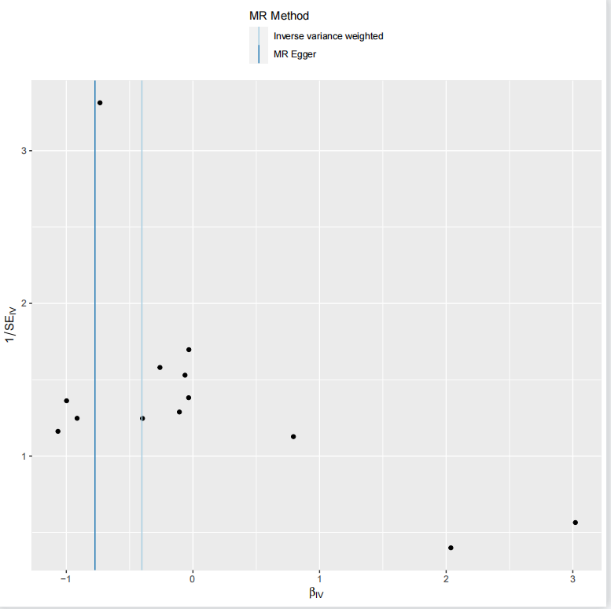

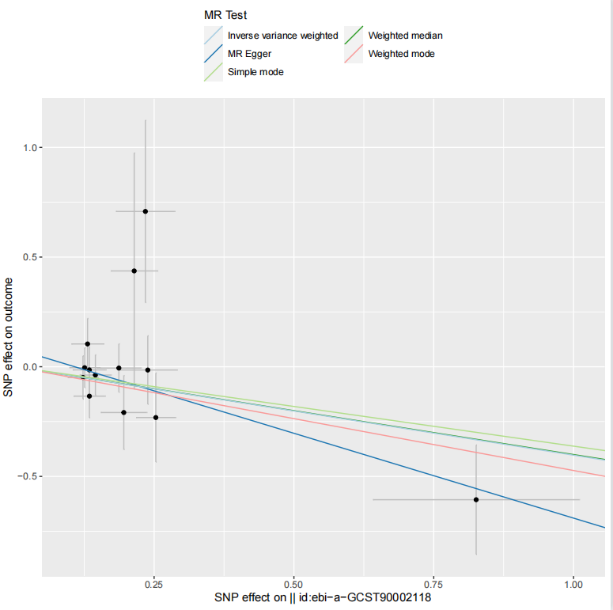


The leave-one-out, funnel, and scatter plots of the causal effect of CD8 on CD28+ CD45RA- CD8br on polymyositis.
