# Supplementary material for: Causality between immunocytes and polymyositis: A Mendelian randomization analysis
Source: Medicine (Baltimore). 2024 Oct 25;103(43):e40254. doi: 10.1097/MD.0000000000040254 (PMC11521033; doi:10.1097/MD.0000000000040254)
Supplement: Supplementary file 4 [file medi-103-e40254-s004.docx]

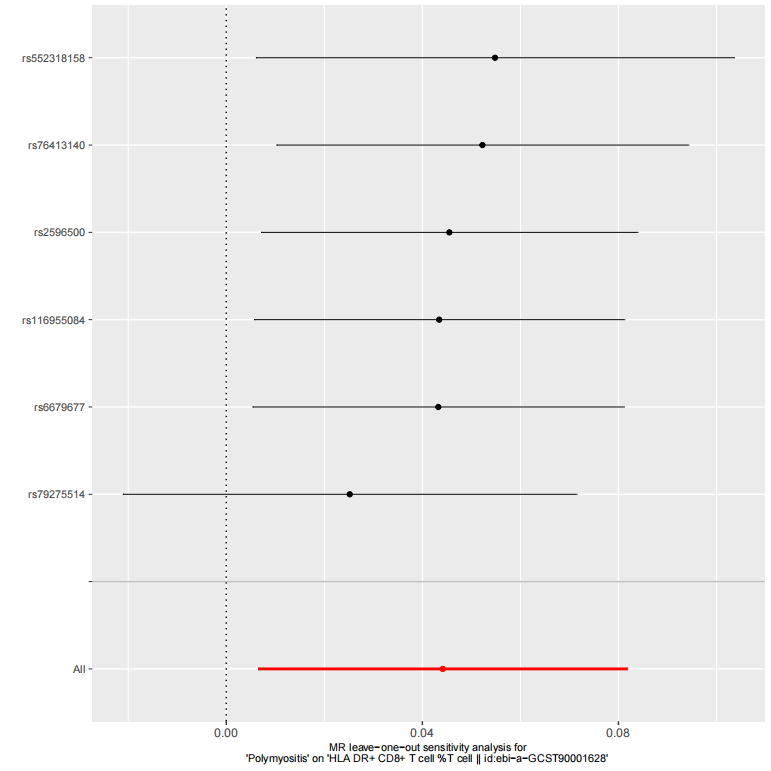

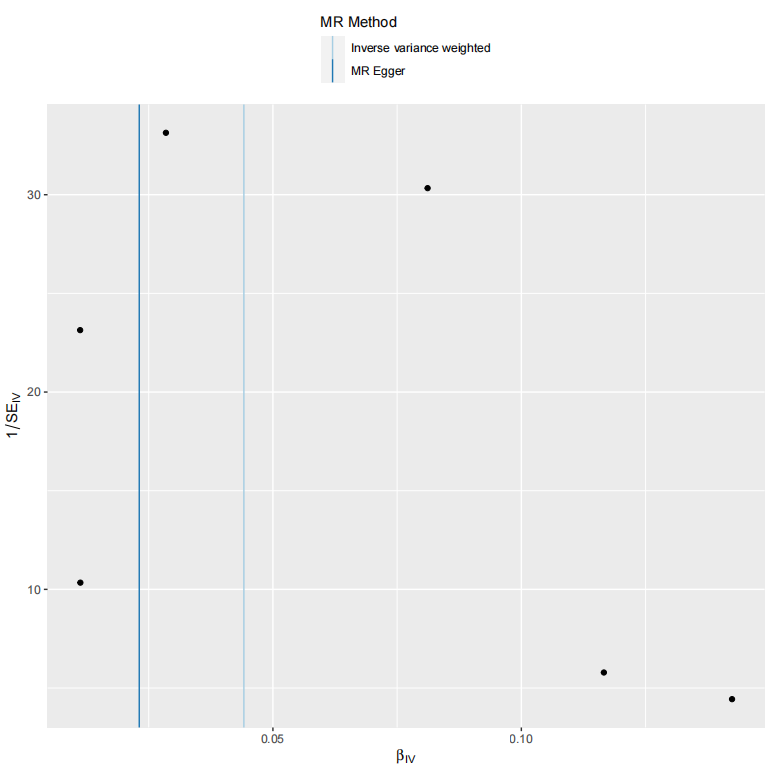

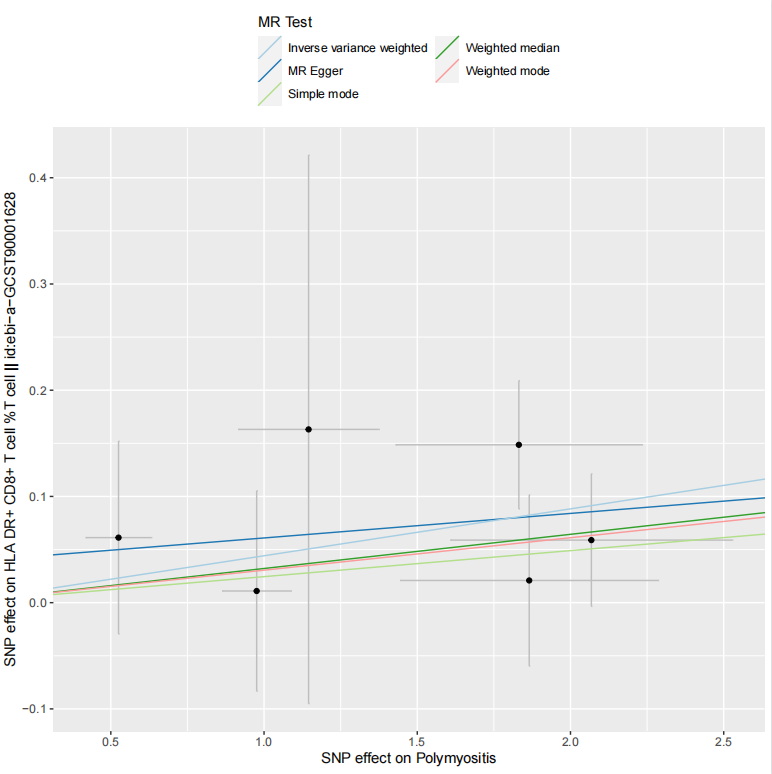


The leave-one-out, funnel, and scatter plots of the causal effect of polymyositis on HLA DR+ CD8+ T cell %T cell.


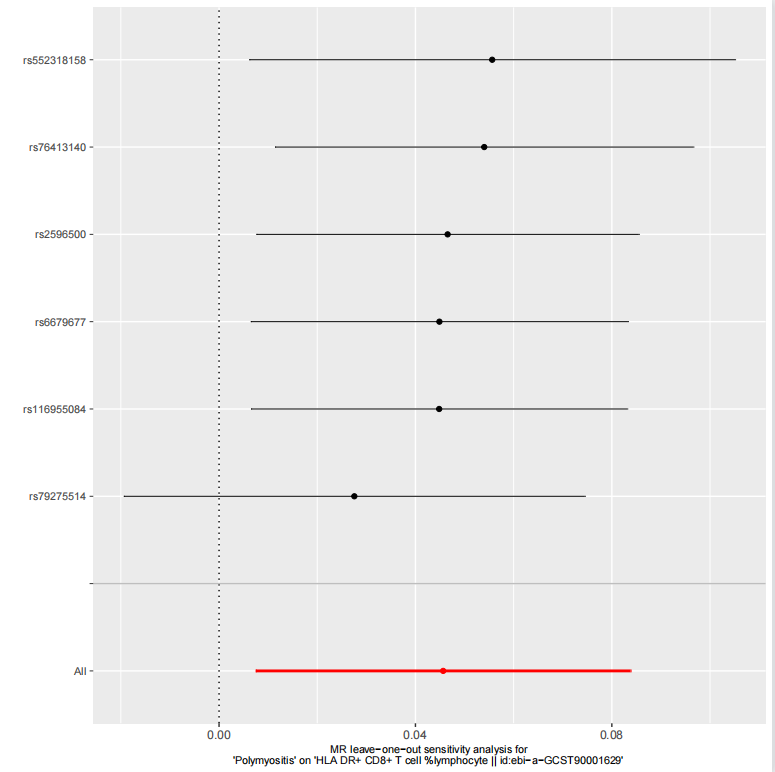


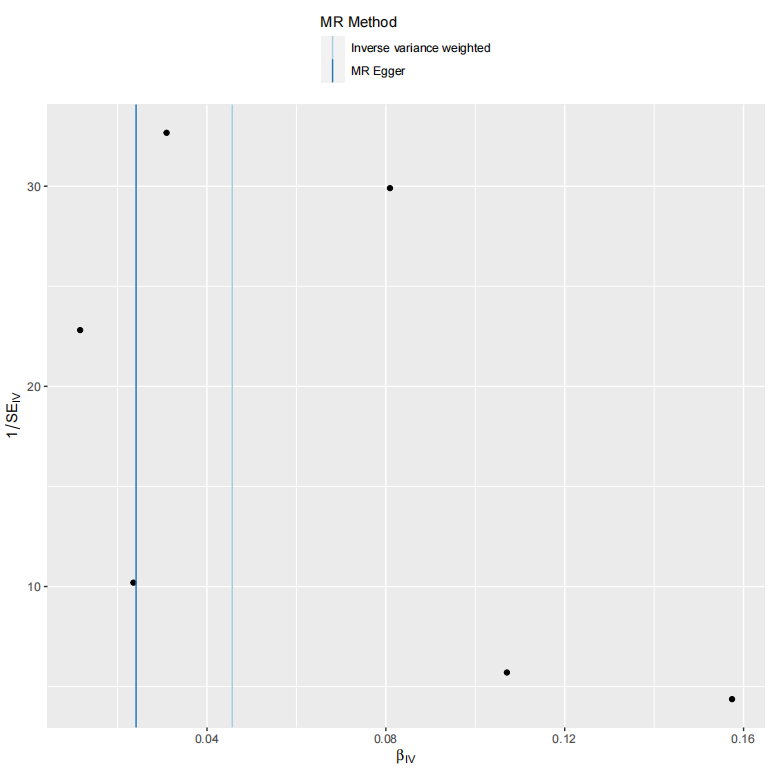

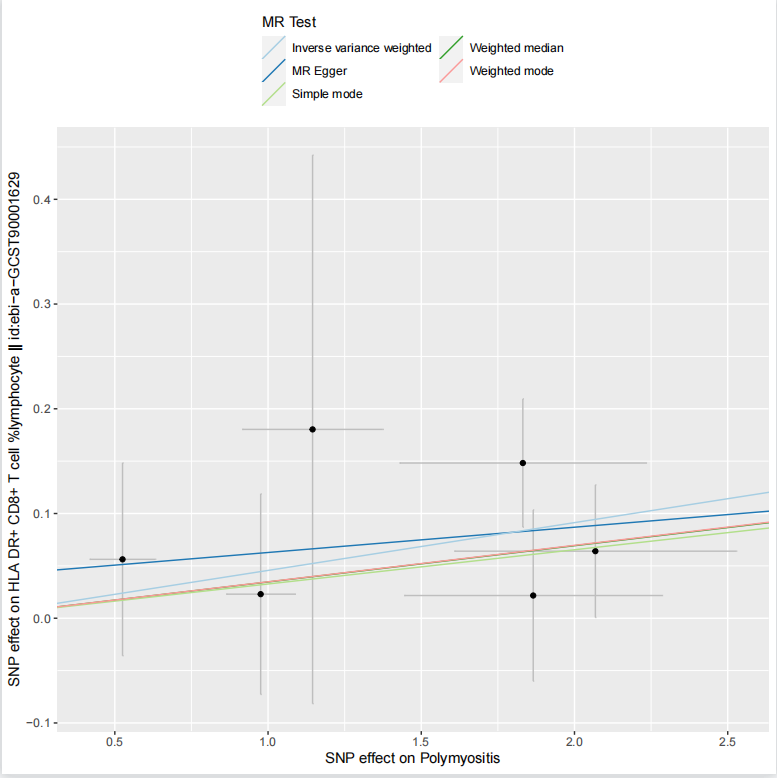


The leave-one-out, funnel, and scatter plots of the causal effect of polymyositis on HLA DR+ CD8+ T cell %lymphocyte.


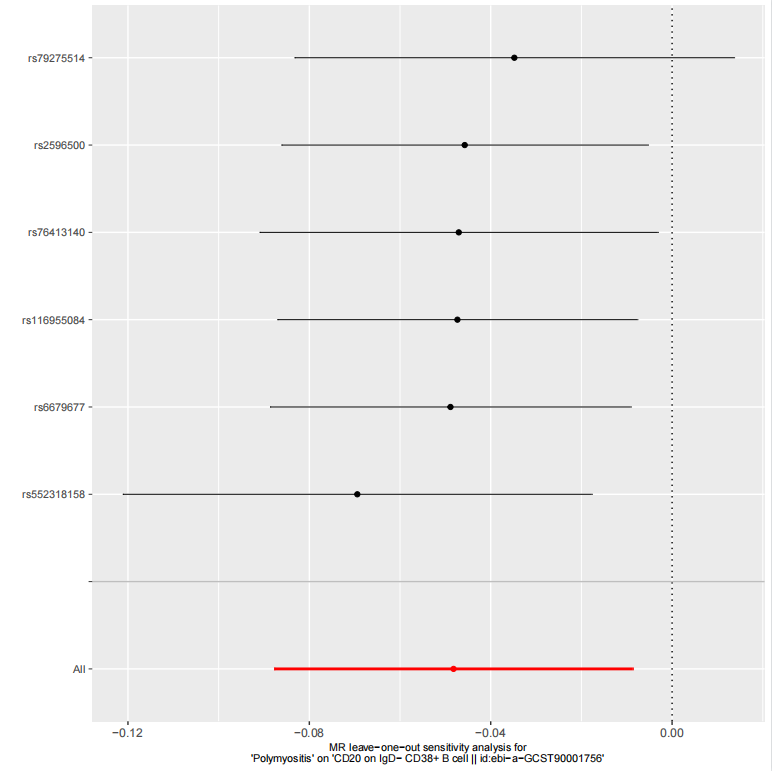


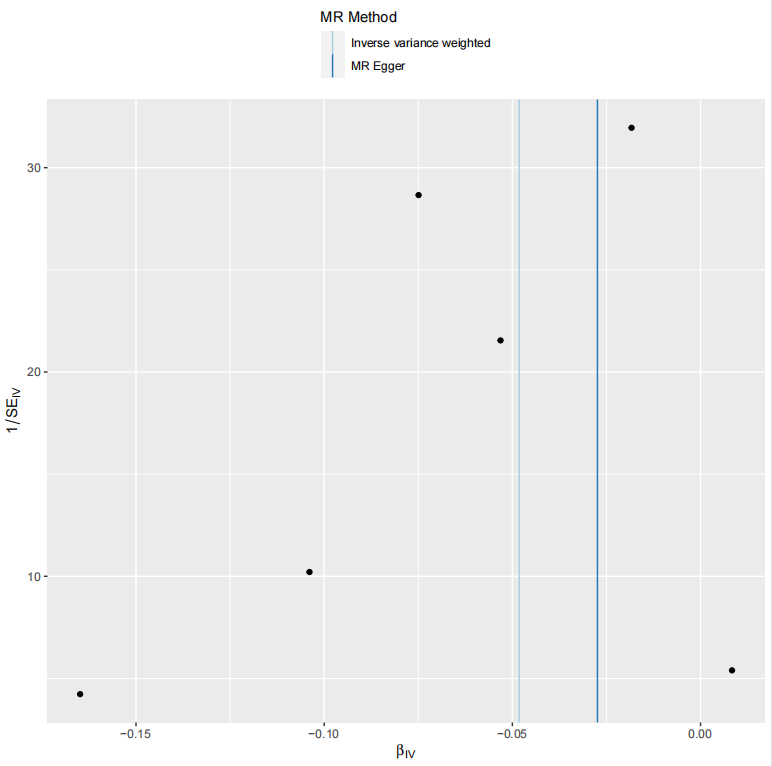

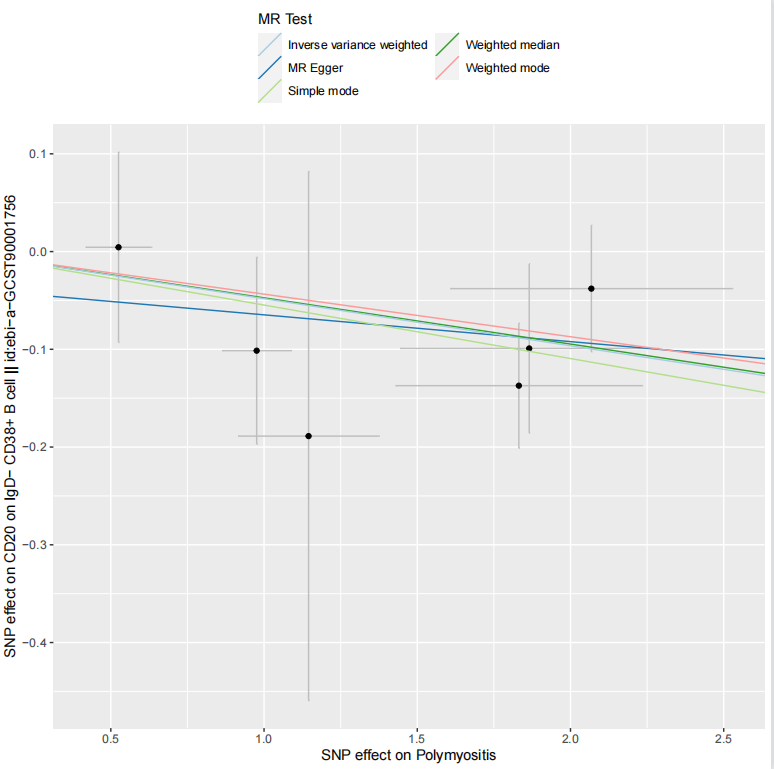


The leave-one-out, funnel, and scatter plots of the causal effect of polymyositis on CD20 on IgD- CD38+ B cell.


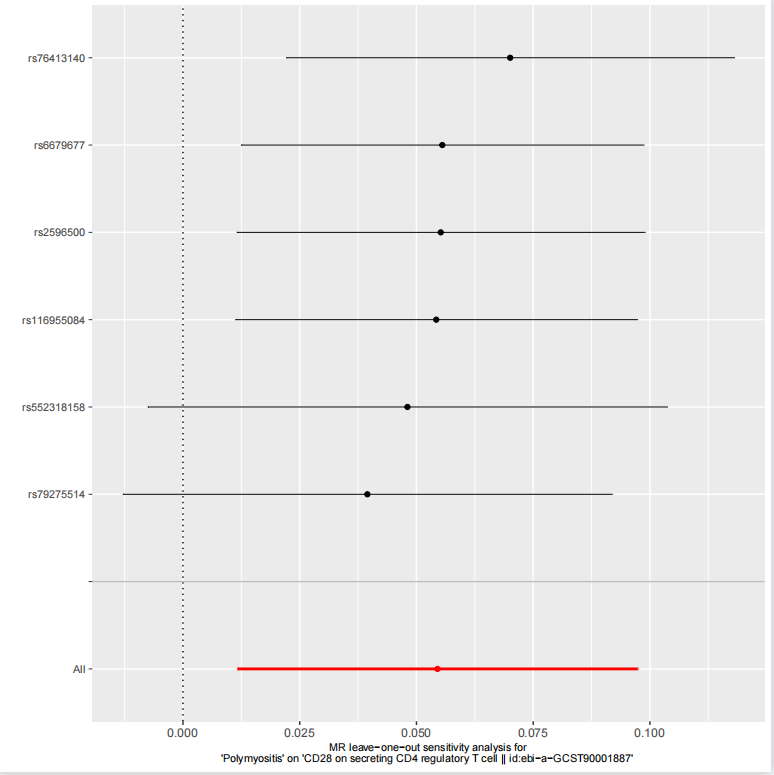


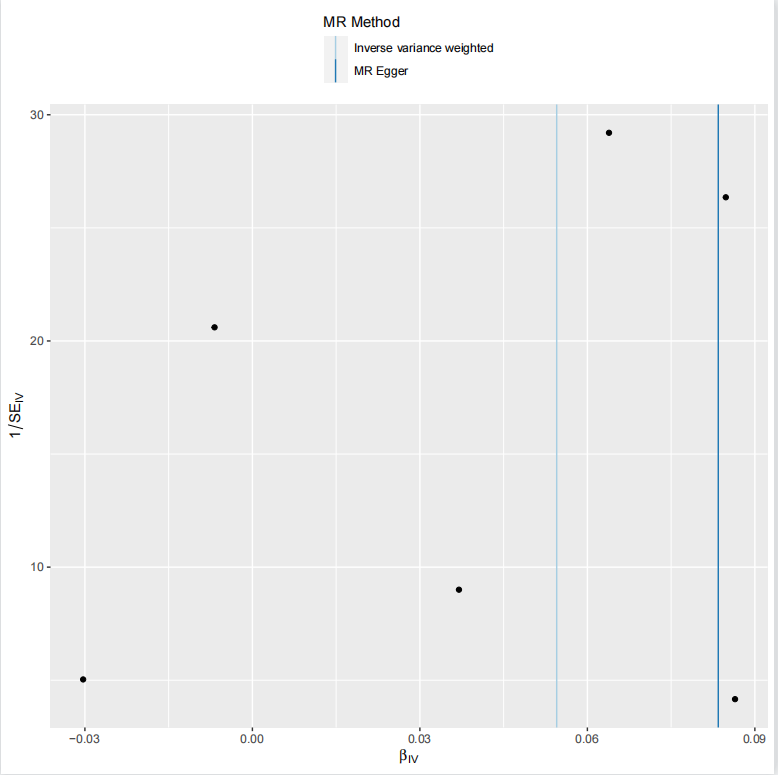

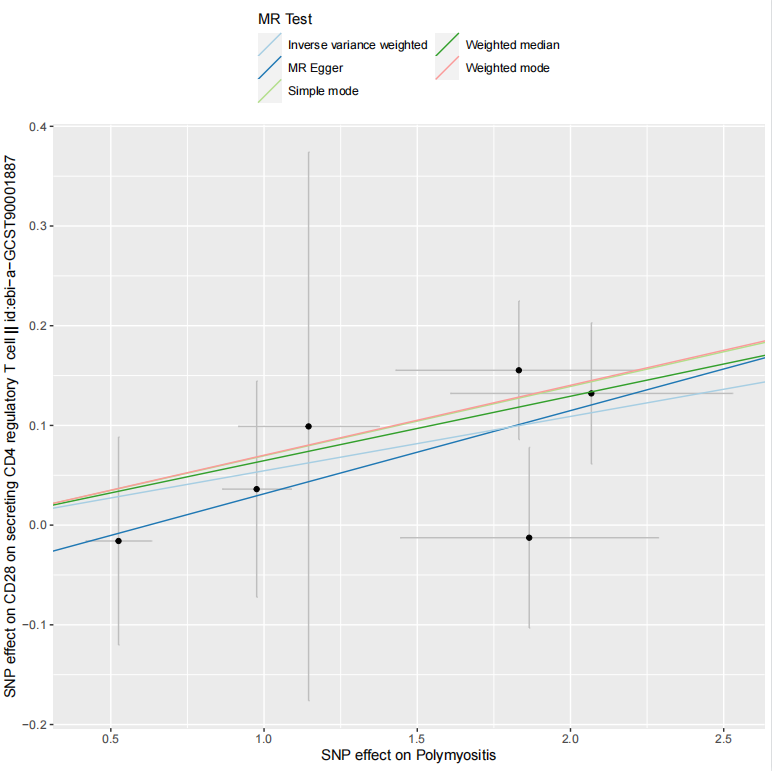


The leave-one-out, funnel, and scatter plots of the causal effect of polymyositis on CD28 on secreting CD4 regulatory T cell.


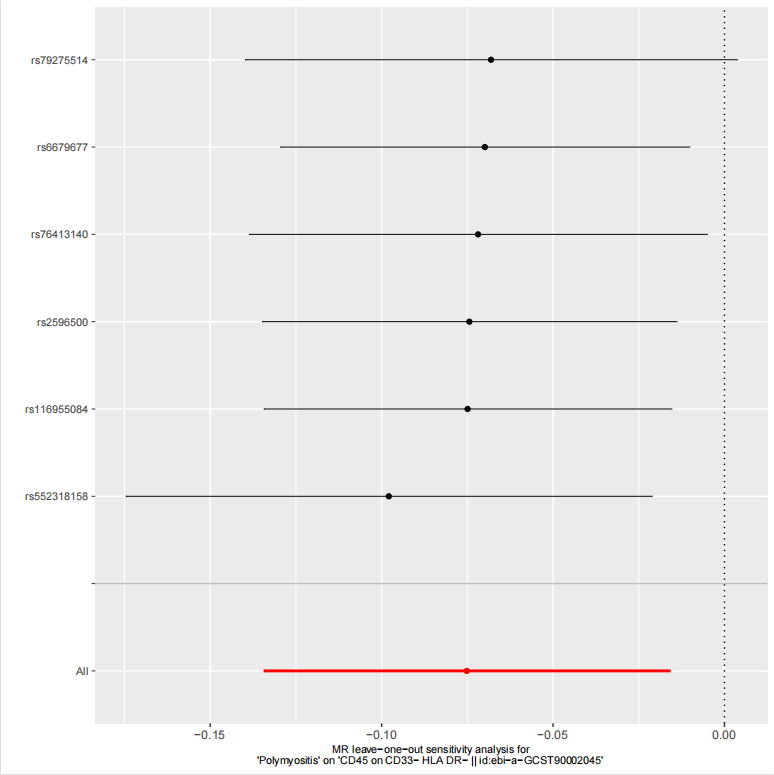


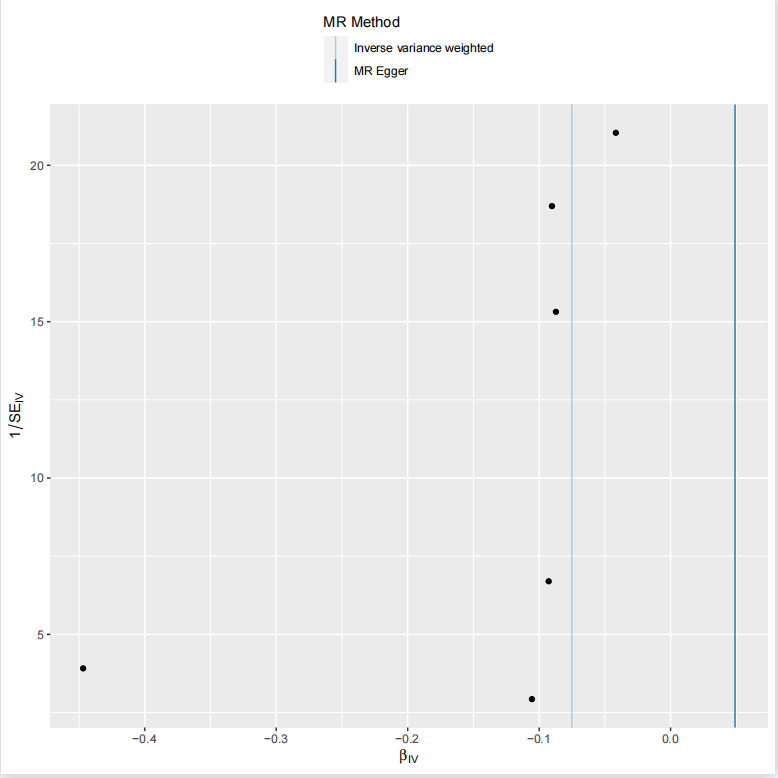

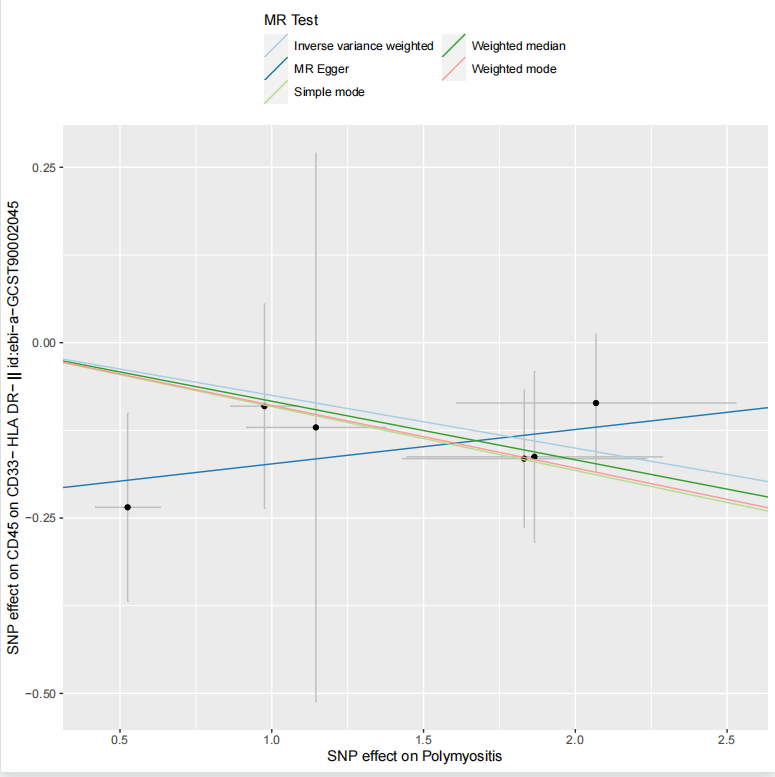


The leave-one-out, funnel, and scatter plots of the causal effect of polymyositis on CD28 on CD45 on CD33- HLA DR-.


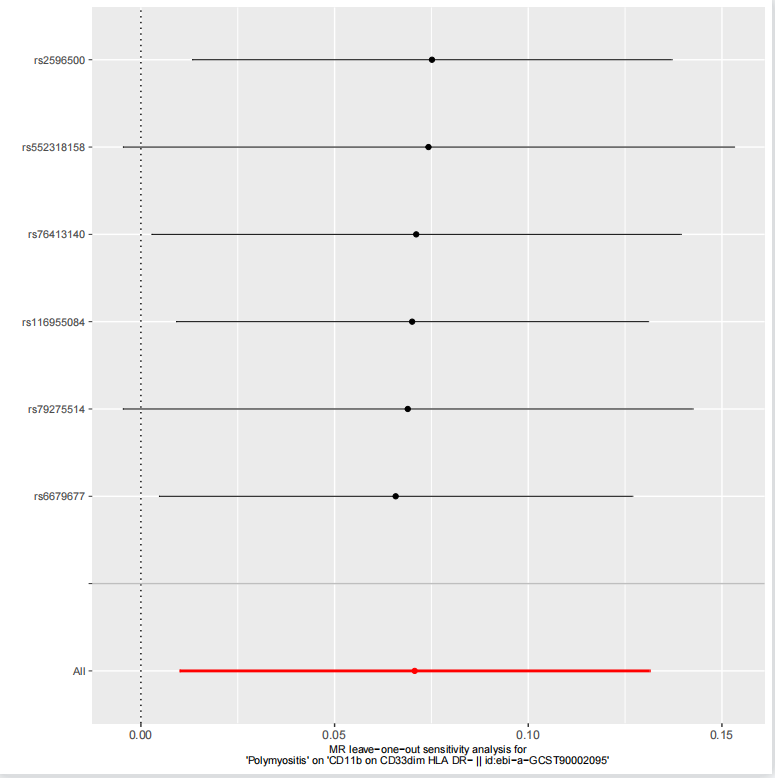


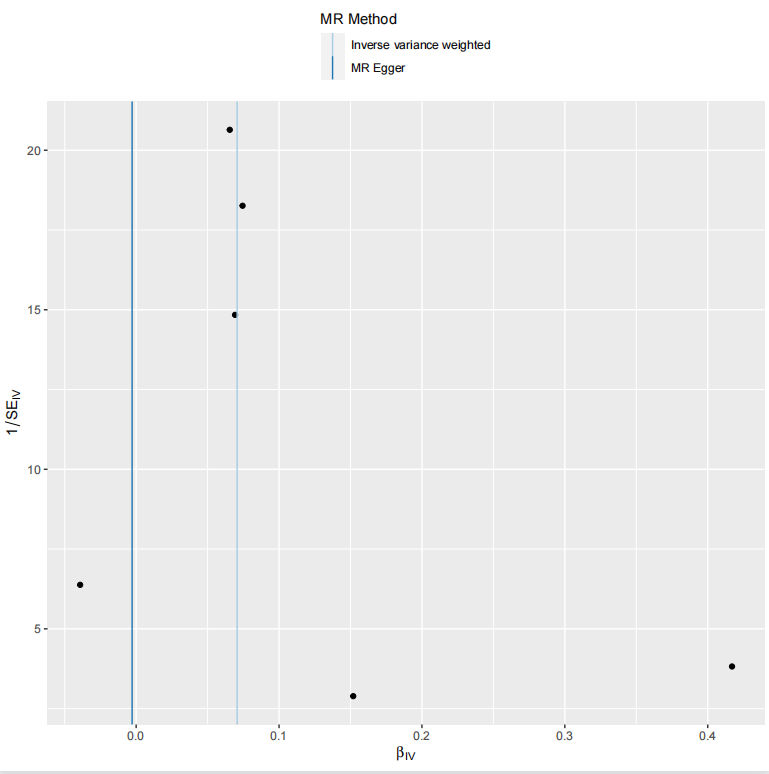

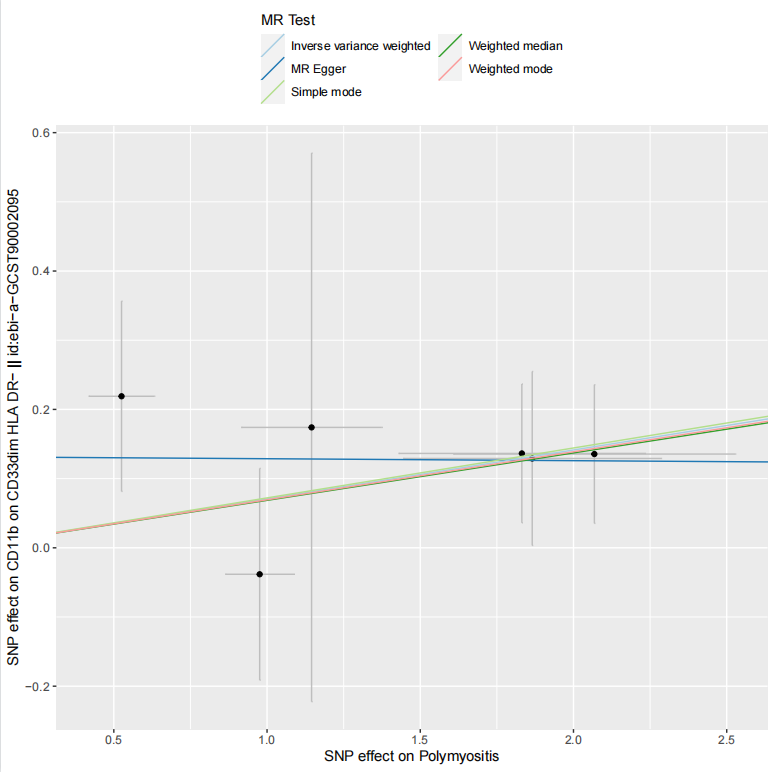


The leave-one-out, funnel, and scatter plots of the causal effect of polymyositis on CD11b on CD33dim HLA DR-.


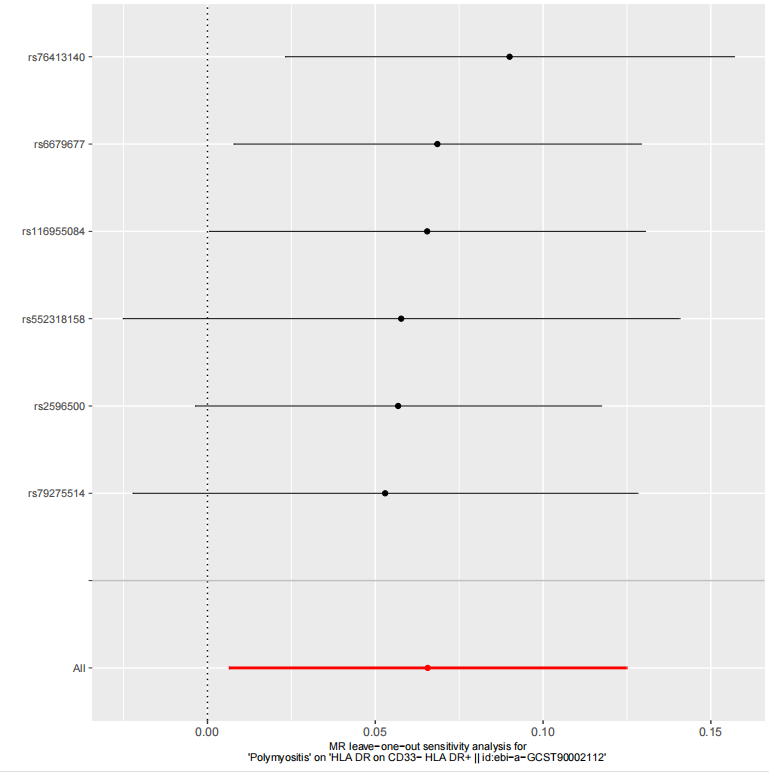


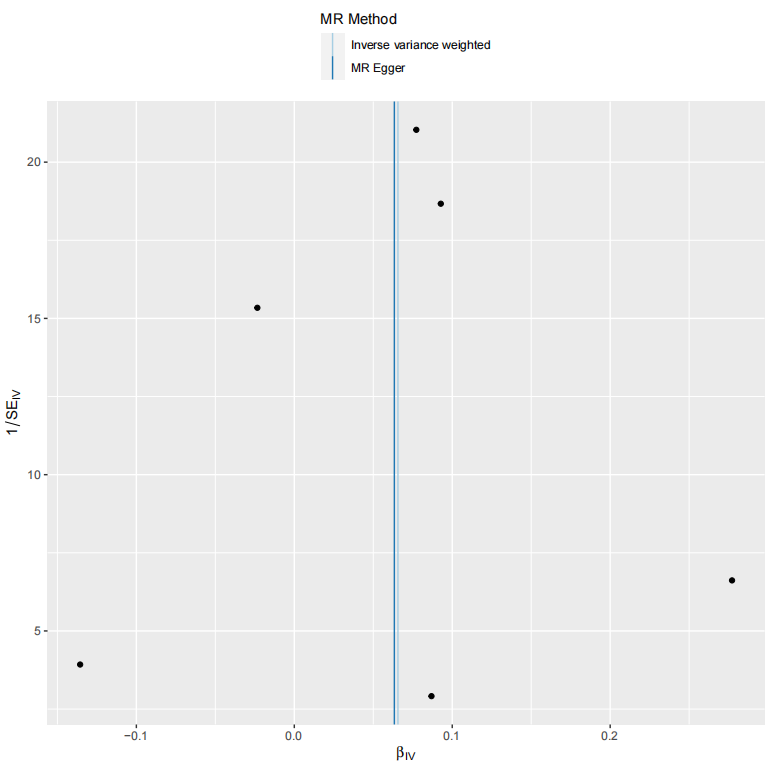

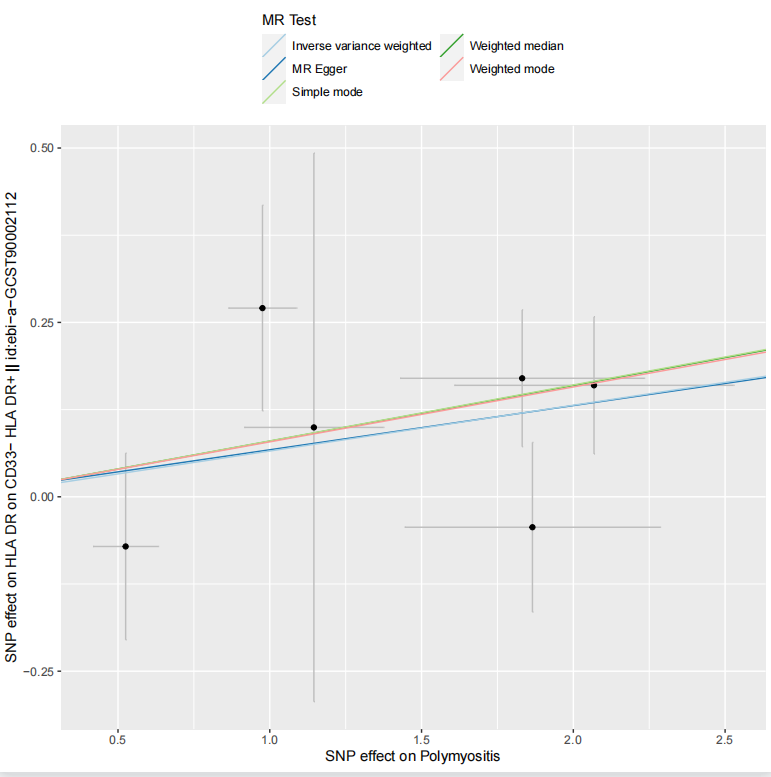


The leave-one-out, funnel, and scatter plots of the causal effect of polymyositis on HLA DR on CD33- HLA DR+.
